# Protocol for a qualitative mechanistic study of MDMA with a sample of psychoanalytic psychotherapists: A phenomenological investigation

**DOI:** 10.1371/journal.pone.0350728

**Published:** 2026-06-18

**Authors:** Elisa Liberati, H. Valerie Curran, Peter Fonagy, Henry Clements, Alessandra Lemma, Rosalind McAlpine, David J. Nutt, Anne Katrin Schlag, Sunjeev K. Kamboj

**Affiliations:** 1 Clinical Psychopharmacology Unit, Research Department of Clinical, Educational and Health Psychology, University College London, London, United Kingdom; 2 Neuropsychopharmacology Unit, Division of Brain Sciences, Imperial College London, London, United Kingdom; 3 Drug Science, London, United Kingdom; Ritsumeikan Asia Pacific University: Ritsumeikan Asia Taiheiyo Daigaku, JAPAN

## Abstract

**Introduction:**

Methylenedioxymethamphetamine (MDMA)-assisted psychotherapy (MDMA-AP) is a potentially transformative intervention for post-traumatic stress disorder (PTSD). Despite encouraging Phase III trial results, the psychological mechanisms underpinning MDMA’s therapeutic effects remain insufficiently understood. Existing research on MDMA has emphasised neurobiological processes but has not adequately incorporated subjective, relational, and experiential processes that are likely to be central to optimising psychedelic-assisted treatments. In the absence of an established clinical psychological theory of MDMA-AP, qualitative approaches can support theory development to refine and optimise MDMA-AP, and identify cross-diagnostic change processes that may broaden its therapeutic scope.

**Materials and methods:**

Approximately 25 experienced psychodynamically trained psychotherapists will receive two doses of MDMA (80–120 mg, oral), separated by ≥1 week, under controlled research conditions in an open-label, qualitative mechanistic study. Clinician-participants will complete baseline interviews examining beliefs and attitudes toward MDMA-AP, brief phenomenological interviews during MDMA sessions, in-depth phenomenological and theory-oriented follow-up interviews examining experience and understandings of MDMA-associated change processes, and daily reflective journals between dosing sessions. Additional standardised measures will assess reflective functioning and related constructs. Qualitative data will be analysed using phenomenological and thematic approaches, and grounded theory-inspired techniques will be used to develop a theoretically coherent model of MDMA’s psychological mechanisms of action grounded in the full dataset.

**Discussion:**

In this study of MDMA’s potential mechanisms-of-action, we treat psychotherapists as expert observers of their own psychological processes. By examining first-hand experience in participants selected for their well-developed capacity for self-reflection, meta-awareness, and advanced theoretical and clinical frameworks for understanding mental phenomena, psychopathology, and psychological treatment, we anticipate generating novel insights into MDMA’s therapeutic mechanisms-of-action. The primary study output will be a theoretically grounded model of MDMA’s psychological mechanisms of action, intended to inform novel treatment models amenable to future experimental evaluation. The study also offers a transferable framework for qualitative mechanistic investigations of psychedelic compounds, supporting the development of integrative and evidence-based models of psychedelic therapy.

## 1. Introduction

Psychedelic-assisted psychotherapy has re-emerged as a highly promising strategy for treating a range of psychiatric disorders [1, 2]. Among psychedelic compounds, the evidence base relating to the entactogen MDMA is particularly advanced, with two Phase III trials supporting its efficacy and safety in post-traumatic stress disorder (PTSD) [3,4]. Yet, despite meeting or exceeding the typical requirements for a treatment to be considered *empirically* *supported*, the US Food and Drug Administration (FDA) did not approve a new drug application for MDMA-assisted psychotherapy (MDMA-AP) in 2024, citing various limitations of the relevant MDMA-AP trials. Additionally, the role of the non-pharmacological components of MDMA-AP and related psychedelic treatments remains uncertain [5,6]. Existing trial designs – which typically compare MDMA + psychotherapy with placebo + psychotherapy – do not disentangle drug from psychotherapy effects. Moreover, because the FDA does not regulate psychotherapy, a key component of the proposed treatment falls outside of current regulatory processes. Additionally, some researchers have questioned the necessity of co-administered psychotherapy in psychedelic assisted psychotherapies [5]. In the case of MDMA-AP, scepticism regarding the psychotherapy component may be driven, in part, by the relative unfamiliarity of psychotherapy researchers with the *inner-directed* *therapy* model used in the main trials [3,4,7], which draws on components of somatic and internal family systems psychotherapies [8–10]. Although other models of psychotherapy have also been proposed as adjuncts to MDMA treatments, these are often narrowly based around cognitive behavioural conceptualisations of psychopathology [11] and continue to focus on PTSD [*cf.* 12]. Indeed, cognitive behavioural therapy has been proposed as a default mode of therapy in psychedelic-psychotherapy combinations by Yaden and colleagues [13]. These authors’ suggestion that cognitive behavioural (and related) therapies should be considered the default, however, relied on somewhat outmoded caricatures of alternative models of psychotherapy (particularly psychoanalysis). Moreover, their suggestions appear grounded in component-level descriptions of therapeutic strategies and their interactions with psychedelics (e.g., *“openness, awareness, and engagement could conceivably potentiate psychedelic sessions”* [13], p.7), rather than process-level mechanistic explanations of how MDMA might interact (additively or synergistically) with these components. Such process-focused explanations might be exemplified by statements like “*MDMA-induced reductions in defensive avoidance may alter the participant’s epistemic stance – for example, by promoting a more trusting attitude towards their therapist – thus encouraging therapeutic engagement and enabling other change processes, like reconsolidation-updating or extinction of traumatic memories”.* In the absence of more precisely specified mechanistic hypotheses of this kind, it is difficult to determine how (or which of) these interactions should be tested experimentally and statistically. As such, we believe the proposal for cognitive behavioural therapy primacy in psychedelic-assisted psychotherapy is, at least, premature.

Current accounts of MDMA’s *psychological* mechanisms of action focus on descriptive behavioural psychopharmacology, for example, its effects on self-compassion, openness, empathy, and fear responding. While these behavioural or subjective variables may form the components (e.g., mediators) of a mechanistic model of MDMA’s therapeutic effects [14], their temporal dynamics (i.e., causal ordering), relational embedding, and interactions with psychotherapeutic support remain underspecified, making it difficult to formulate discriminating predictions.

To summarise, current descriptions of MDMA-induced psychological phenomena remain theoretically underdeveloped and insufficiently integrated into existing theories of psychological change. The lack of a shared lexicon or validated process measures further contributes to the conceptual gaps between descriptive behavioural-psychopharmacological findings, process-oriented psychotherapeutic studies and clinical trial research. Without a well-developed understanding of MDMA’s mechanisms of action, it is difficult to establish which non-pharmacological elements of MDMA-AP are most essential, how these elements might be prioritised and optimised, and which elements (if any) might be unhelpful or even harmful (including, but not limited to, psychotherapeutic modality, relational stance, exposure to traumatic material, dosing-day support, as well as preparation provision and post-dosing meaning-making). These questions are, of course, based on the assumption that at least *some* psychotherapy is needed in MDMA treatment, which, as noted above, remains a contentious point in psychedelic medicine research [15].

For this study, we devised an innovative qualitative protocol aimed at developing a theory of MDMA’s therapeutic mechanisms of action by investigating its effects in a specific participant group: psychotherapists with verified credentials in psychoanalytic/psychodynamic psychotherapy. Our participants will have limited past experience with MDMA and will be administered the drug in a controlled research setting under clinical supervision. Due to their training – a compulsory component of which is completion of extensive personal therapy – these participants are likely to be highly skilled in self-reflection, to possess an advanced capacity for describing their own cognitive and affective processes, and to be able to apply theoretical frameworks to understand mental phenomena in general, and psychopathology and its treatment in particular. The detailed qualitative exploration of participants’ first-hand experiences of MDMA will, therefore, provide a credible, clinically informed basis for developing a psychological model of MDMA’s action.

The objectives of this study are, therefore:

1. To explore the phenomenology of MDMA experience in psychodynamic psychotherapists under controlled research conditions.
2. To examine subjectively experienced changes in self-experience and relational patterns.
3. To invite participants to frame these experiences in terms of psychological concepts relevant to theory-building of MDMA–psychotherapy combinations (participants’ spontaneous accounts and theory-oriented formulations will be treated as conceptually distinct and analysed separately).
4. To synthesise insights into a theoretically grounded model of MDMA’s therapeutic mechanisms of action (ideally linking qualitatively generated experiential sequences to operationalisable constructs) and to generate testable hypotheses (e.g., about mediation, moderation and causal pathways) for future clinical and experimental trials.

To simulate aspects of the use of MDMA in recent clinical trials [1], participants will receive up to 120 mg MDMA in each of two dosing sessions, separated by at least one week. No formal psychotherapeutic procedures (e.g., those intended to direct trauma or experience processing) will be employed.

The study objectives will be achieved through a series of in-depth interviews, narrative journaling, and other psychological assessments before and after the dosing sessions. In addition, brief interviews will be performed *during* each dosing session. Qualitative data will be analysed using phenomenological approaches and thematic analysis to examine changes in participants’ attitudes, self-experience, emotional processing, and relational patterns across baseline, acute and sub-acute (dosing sessions) phases and follow-up phase. The qualitative findings will be integrated with other data sources (journalling and validated questionnaires) using techniques inspired by grounded theory (such as the constant comparison approach [16]), with a view to producing a theoretically rigorous model of MDMA’s psychological mechanisms of action.

## 2. Materials and methods

### 2.1 Study design

This is an open-label, single-site, qualitative mechanistic study conducted at an academic psychopharmacology facility. The study has received ethical approval from University College London’s Research Ethics Committee (REC reference 2025-0272-336).

### 2.2 Sample and recruitment

#### 2.2.1 Rationale for the psychodynamic orientation.

We will recruit trained psychodynamic psychotherapists who meet the eligibility criteria. The clinical approach of psychodynamic clinicians is grounded in psychoanalytic theory and emphasises the role of unconscious processes, early relational experiences, and internalised attachment orientations in shaping current thoughts, affect, and behaviours. Central to this orientation are the concepts of transference and countertransference [17]. The latter refers to the therapist’s emotional responses to the patient/participant, which are viewed as an invaluable source of information about the patient/participant’s internal world. Effective psychodynamic practice, therefore, requires therapists to cultivate self-reflective capacities and a willingness to explore their own inner world, so that countertransference can be recognised and used constructively within the therapeutic relationship. This emphasis on self-awareness is reflected in lengthy clinical training that, in combination with mandated personal psychotherapy (usually comprising at least two sessions weekly for several years), is believed to increase meta-awareness and the facility for describing mental states [18,19]. In this respect, the psychodynamic orientation departs from other psychotherapies that are more focused on behavioural change (such as cognitive behavioural therapy) or on empathy and unconditional positive regard (such as humanistic therapy), for which personal therapy is typically not mandated. Our decision to focus on psychodynamic psychotherapists precisely reflects the need for our participants to be highly skilled in self-reflection and to possess a language for describing subjective experience with precision.

Although this methodological choice presents other limitations – primarily that participants may be primed to interpret their experiences through a particular theoretical lens – our analysis will deliberately separate description of first-hand experience from interpretation (these will be elicited through different instruments and at different time-points; see section 3).

#### 2.2.2 Eligibility criteria.

As a starting point, inclusion criteria are as follows:

- Age 25–65 years
- Qualification in psychodynamic psychotherapy obtained through a British Psychoanalytic Council (BPC) or United Kingdom Council for Psychotherapy (UKCP) accredited institution
- Personal therapy during training comprising ≥2 sessions weekly, delivered by a psychoanalyst or a psychodynamic psychotherapist
-  ≥ 5 years post-qualification

Exclusion criteria include:

- MDMA use in the past five years
- Regular psychedelic use in the past
- Psychiatric disorder
- Use of psychotropic medication
- Metabolic or cardiovascular disease
- Neurological conditions
- Pregnancy or breastfeeding
- BMI < 18 or >30 kg/m²
- Any abnormal finding at the health assessment

We aim to recruit approximately 25 individuals. The sample size is informed by the concept of *information* *power* [20], which suggests that the more information a sample holds that is relevant to the study aim, the fewer participants are required. Given the multiple data-collection points per participant, the diversity of data sources (interviews, questionnaires, journals), and the specific characteristics of the targeted population, we anticipate that a pragmatic sample size of approximately n = 25 will generate sufficient data to meet the study aims, reach meaning saturation [21], and permit meaningful comparisons across participants without incurring data waste.

Recruitment will be via British Psychoanalytic Council registers, training institutions, specialist conferences, professional networks, and word of mouth. It is possible that some of the above criteria (other than those required for safe drug administration) may need to be relaxed if recruitment efforts indicate they are overly restrictive. Any changes to eligibility criteria or other aspects of the protocol described here will be reviewed through a formal amendment to the Research Ethics Committee and implemented only following their approval.

### 2.3 Study procedures

**Fig 1:**
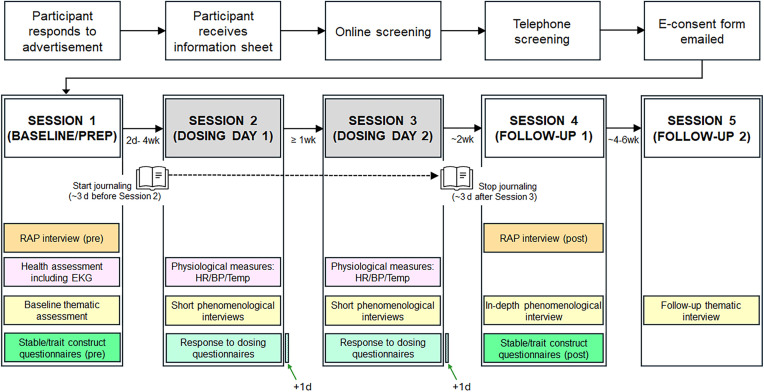
Study flowchart. Session timelines and assessments. RAP: Relational Anecdote Paradigm. Physiological measures will consist of EKG (electrocardiograph) at baseline and periodic HR (heart rate), BP (blood pressure) and Temp (skin temperature) on Sessions 2 and 3. Periodic assessment of subjective drug effects will use the Drug Effects Questionnaire (DEQ) and Positive and Negative Affect Schedule (PANAS) short-form. Note: The possible additional micro-phenomenology interview (potentially in Session 4) is not shown.

Eligible participants who decide to take part will provide written informed consent in line with the principles expressed in the Declaration of Helsinki. Participants will then complete five sessions plus longitudinal journaling (see Fig 1).

- **Session 1 (Baseline data collection and preparation for dosing).** After the online screening and follow-up telephone pre-screening, Session 1 includes an additional health screening and final confirmation of eligibility. Baseline psychological questionnaires, baseline Relational Anecdote Paradigm (RAP) interview, baseline thematic interview, and preparation for the dosing sessions will also be completed on Session 1. The health screening includes a 6-lead EKG and blood pressure assessment. Findings will be reviewed by a medical doctor or a research nurse prior to participation in Session 2 (first dosing session). Given the multiple data collection points in this session, researchers will ensure flexible timing and plan breaks to reduce participant burden. Preparation for the dosing session covers ‘setting’, explanations about the psychological and physiological effects of MDMA, timing of phenomenological interviews, and agreements concerning music, use of eyeshades, and touch (limited to participant-initiated hand-holding).
- **Sessions 2 and 3 (Dosing and acute/sub-acute data collection)**: These sessions include oral administration of 80–120 mg MDMA, physiological monitoring, acute-phase monitoring questionnaires, and brief phenomenological interviews during the acute and sub-acute exposure phases. Our decision to include two dosing sessions was informed by a desire to simulate the multiple dosing typically used in clinical trials of MDMA-AP (typically three dosing sessions, e.g., [3,4]), while ensuring participant burden is minimised.

During dosing, the researchers’ stance will be supportive and non-directive. Support will comprise physiological monitoring, response to practical needs, verbal reassurance, and, at the participant’s request, basic grounding techniques such as guided slow breathing or hand-holding, intended to manage acute episodes of distress or fulfil participants’ expressed need for safety or comfort. If distressing material arises spontaneously, researchers will acknowledge this with empathy and warmth, without interpretation, reappraisal, or any other psychotherapeutic technique. Participants will be gently redirected to present-moment phenomenology.

**Sessions 2 + 1 day and 3 + 1 day (Post-acute data collection)**: These assessments are designed to be relatively low-burden question(naire)s completed online. Except for an assessment of current affect, the question(naire)s refer to participants’ retrospective response to the dosing session (see Table 1).

**Table 1:** Candidate psychological constructs (elements of candidate mechanisms of action) and corresponding data collection tools.

Phase	Relevant psychological construct	Data collection method
Baseline	N/A	General thematic interview (prior attitudes, beliefs and ideas regarding MDMA and MDMA-AP).
Acute and sub-acute (dosing session)	Embodied experience/shifts	Brief phenomenological interviews. Drug Effects Questionnaire (DEQ). Physiological measures.
	Cognitive experience/shifts	Brief phenomenological interviews.
	Affective experience/shifts	Brief phenomenological interviews. Positive & Negative Affect Schedule (PANAS-SF).
	Imagery experience/shifts	Brief phenomenological interviews.
Post-acute (1d post dosing)	Affective experience/shifts	PANAS-SF.
	Response to music (during dosing)	Geneva Emotional Music Scale. Bespoke music-related ratings.
	Self-related processes	Sense of Self Scale.
	Emotion confrontation/resolution	Emotional Breakthrough Inventory (EBI).
	Altered consciousness	5-Dimensional Altered States of Consciousness Scale (5D-ASC).
	Challenging experiences	Challenging Experiences Questionnaire (CEQ).
Follow-up	Self-related processes	In-depth phenomenological interview. Relational Anecdote Paradigm (RAP) interview. Sussex-Oxford compassion scales (SOCS-Self).
	Self-other processing and relational experiences	In-depth phenomenological interview. RAP interview. SOCS-Other. Epistemic Trust Questionnaire (ETQ-15). Experience Close Relationships Scale (ECR-36). Inventory of Interpersonal Problems (IIP-32).
	Epistemic stance	ETQ-15.
	Reflexive functioning	RAP interview.
	Defence mechanisms	In-depth phenomenological interview. RAP interview.
	Compassion (towards self & other)	SOCS-Self and SOCS-Other.
	Empathy	Toronto Empathy Questionnaire (TEQ).
	N/A	Follow-up thematic interview (post-experience theoretical integration).

- **Session 4 (Follow-up data collection)**: This session, conducted remotely (via videoconferencing), includes a follow-up RAP interview, psychological questionnaires, and an in-depth qualitative interview. If a micro-phenomenological interview is included, it will take place on Session 4.
- **Session 5 (Follow-up data collection)**: Like Session 4, this session is conducted remotely and includes a follow-up thematic interview inviting theoretical integration of the MDMA experience and views on its mechanism of action in MDMA-AP. The interview will also cover participants’ perspectives on music during their experience, and more broadly, in MDMA-AP.
- **Journaling**: Participants will be asked to prepare brief daily written or audio reflections from three days before Session 2 until three days after Session 3.

### 2.4 Medicinal product

MDMA is provided by PharmAla Biotech (Canada) in 40 mg capsules. Based on recommendations from recent clinical and experimental studies of MDMA, we will administer a total dose of up to 120 mg per session on two sessions separated by ≥1 week (see also Section 2.7 for rationale for the dose). As is typical of clinical trials, an initial 80 mg dose will be followed by a *top-up* dose of 40 mg after approximately 90 minutes if the participant consents and tolerates the lower dose well. This split-dosing protocol extends the duration of action of MDMA while avoiding a rapid peak effect (as might be expected from a 120 mg bolus). If the participant does not wish to proceed with the top-up dose, or if there are concerns about tolerability in either dosing session, the lower (80 mg) will be the final recorded dose for that session. This flexibility in dosing was deemed appropriate by the FDA in the two largest Phase III clinical trials of MDMA for PTSD, in recognition of uncertainty regarding the optimal dose [1].

Participants’ physiological and psychological responses will be closely monitored throughout the dosing session (approximately 8 h). Harmful or unpleasant responses will be monitored and documented throughout the dosing session. Attributability of these responses to MDMA will be determined by the supervising medical professional and categorised as mild (no impact on daily activities), moderate (some interference with daily activities), or severe (prevents daily activities), and as anticipated or unanticipated.

Drug transport, handling, and storage procedures will comply with the conditions set out under the research unit’s Schedule I controlled drug licence granted by the relevant UK government department (the Home Office). Access to the controlled-drug storage facility at University College London is strictly controlled, and robust documentation procedures for drug handling are in place and subject to regular Home Office inspections.

### 2.5 Data collection tools

#### 2.5.1 Qualitative interviews.

We will use several forms of semi-structured qualitative interviews to capture participants’ experience-near descriptions of MDMA effects, reflective meaning-making, and theory-driven interpretations at different points across study phases, as outlined in Table 1.

A) **Phenomenological interviews** will be used to explore participants’ first-hand experience and sense-making of the dosing sessions.
  - **Brief phenomenological interviews** (10–20 min) are planned during each dosing session at approximately t = 1.5 h, 3 h, and 5 h, and at the end of the session (t ≈ 7 h). These interviews will encourage participants to attend to their experience in the here-and-now and to describe changes in thoughts, feelings, bodily sensations, embodied experience, and mood as drug effects unfold. The frequency and timing may be adjusted based on early participants’ feedback and acceptability; for example, the number of interviews may be reduced.
  - An **Interpretative Phenomenological Analysis (IPA) interview** [22] will be conducted in Session 4 (after completion of the second dosing session) to elicit participants’ experience and meaning-making of the MDMA sessions. As with the brief phenomenological interviews, the focus will remain on experience-near accounts, and participants will also be invited to elaborate their interpretation and meaning-making of the MDMA experiences. The interviewer will additionally explore changes in specific domains, including sense of self and self–other experience.
  - A **micro-phenomenological interview** [23] may be incorporated into Session 4 for some participants, depending on whether preliminary findings suggest that this additional detailed procedure is justified (i.e., is not excessively burdensome and is likely to yield significant additional phenomenological insights not obtained from IPA interviews). This method supports heightened awareness and precise description of a re-evoked experience. Rather than structuring the exchange around topic-based questions, the interview aims to orient participants’ attention to moment-to-moment features of the re-evoked event. Here, the objective is to obtain a detailed account of the moment participants become aware of MDMA effects, focusing on the procedural, embodied dimension of experience. This approach is intended to identify aspects that may remain implicit or unfold at a pre-reflective level.
B) **A modified Relationship Anecdote Paradigm (RAP) interview** will be conducted in Sessions 1 and 4. Participants will be asked to recall a small number of interactions between themselves and another person (including friends, primary caregivers, and their psychotherapists). For each interaction, participants will be asked contextual questions (e.g., when, with whom), questions about their own and the other’s statements, behaviours, and reactions, and to describe how the episode concluded. At the end of the exercise, participants will be asked to recall two events that are memorable because of their characteristics: one described as an unpleasant interaction that they sometimes replay and wish had not happened, and one described as a pleasant interaction that forms a happy memory.

The original RAP was devised to support analysis of Core Relationship Conflict Theme (CCRT) and has been described as the *first operational* *method* for assessing transference [24]. From a psychodynamic perspective, CCRT reflects a relational template forged in early infancy that informs subsequent close relationships; thus, assessing CCRT at different time points provides one method for examining shifts in well-established relational patterns. RAP interviews have been used in numerous studies and adapted to specific aims, including examination of therapeutic closeness and distance and changes in the therapeutic relationship in relation to outcomes [25,26]. In the present study, RAP material will support examination of changes in reflective functioning [27], relational patterns (via CCRT), and, qualitatively, changes in the content and quality of recalled episodes before and after MDMA exposure.

C) **Thematic interviews** will explore participants’ knowledge of, and attitudes toward, MDMA-AP and MDMA as a potential therapeutic agent. These interviews will be conducted prior to and after the dosing sessions (Sessions 1 and 5) to evaluate changes in attitudes and understanding. In Session 5, the thematic interview will also invite participants to conceptualise their first-hand MDMA experiences using psychological concepts relevant to theory building and intervention development.

#### 2.5.2 Questionnaires.

Stable traits will be assessed using validated questionnaires administered during Sessions 1 and 4 to evaluate changes in: compassion toward self and others (Sussex-Oxford Compassion Scales (20 items each for self- and other-) [28]), empathy (Toronto Empathy Questionnaire [29]), epistemic trust (The Epistemic Trust Questionnaire (ETQ-15) [30]), attachment patterns (Experiences in Close Relationships Revised Scale (36 items) [31]), and interpersonal problems (Inventory of Interpersonal Problems (IIP, 32 items) [32]).

Acute subjective drug response will be assessed using two brief self-report ‘response to dosing questionnaires’ (Fig 1): the Positive and Negative Affect Schedule-Short Form (PANAS-SF; [33]) and the Drug Effects Questionnaire (DEQ; [34]) at t = 0 (pre-drug baseline) and t = 0.5 h, 1.5 h, 3 h, 4.5 h, and 5.5 h post-drug on each dosing session.

A post-acute evaluation of participants’ response to the dosing session will be conducted the day after each dosing session remotely via an online survey. Apart from repeating the PANAS-SF, which refers to *current* affect, the other question(naire)s completed on Session 2 + 1d and Session 3 + 1d refer to participants’ experiences of the dosing procedure: Emotional Breakthrough Inventory (EBI: [35]), 5-Dimensional Altered States of Consciousness (5D-ASC; [36]), Challenging Experience Questionnaire, 7-item [37] and Geneva Emotional Music Scale [38]. Additional bespoke ratings will be obtained regarding the interaction between the music and MDMA.

#### 2.5.3 Physiological measures.

Non-invasive measures of blood pressure, heart rate, and body temperature will be taken at intervals during dosing sessions.

#### 2.5.4 Reflective journals.

Participants will complete a brief daily journaling task (via a bespoke bot designed for this study, on a secure mobile app) during their involvement in the research. They will be asked to provide a reflective overview of the day’s thoughts and feelings (the task should take no longer than 3–5 minutes daily). Depending on the interval between Sessions 2 and 3, participants will journal for a minimum of 13 days and a maximum of 20 days.

### 2.6 Data analysis plan

#### 2.6.1 Interviews.

All interviews will be recorded and transcribed verbatim. The analysis may be supported by software such as QSR NVIVO.

A) **Phenomenological interviews.** These will be conducted as experience-near brief interviews during dosing sessions, as well as more in-depth interviews at follow-up. The interviews will be analysed using IPA principles as outlined by Smith and colleagues [22]. IPA is committed to in-depth examination of lived experience, with a focus on participants’ meaning-making and their own language. Following interview transcription, an experienced qualitative researcher will undertake exploratory noting to capture key content and highlight passages that are particularly rich in meaning. Provisional interpretative notes will be developed and iteratively queried during analysis. The researcher will then generate experiential statements, through which initial open interpretations are refined and stabilised. These will be organised thematically and clustered into Personal Experiential Themes. Each participant’s interviews will first be analysed ideographically across time-points to develop a rich account of subjective experience of MDMA as it unfolds over time. These accounts will then be synthesised to generate Group Experiential Themes, integrating patterns across the full qualitative dataset. It is expected that analysis of the brief interviews (conducted during the dosing sessions) will foreground immediate experience over meaning-making, with results being used primarily for temporal anchoring and capturing evolving experience. The analytical focus of the in-depth IPA interviews will be on meaning-making and idiographic depth, as well as experience.
B) **Micro-phenomenological interviews:** If micro-phenomenology interviews are included (see Section 2.5.1), the focus of enquiry and analysis will be on fine-grained phenomenological events and processes at specific moments. In line with the approach proposed by Petitmengin [23], analysis will identify recurring experiential patterns or *microstructures* within participants’ narratives. Transcripts will be segmented into experiential units and examined for sensory modalities, emotional tone, attentional focus, and implicit dynamics. These units will then be abstracted into models or maps of experience.
C) **Thematic interviews:** Using thematic analysis [39], the focus will be on attitudes toward MDMA-AP, changes in such attitudes, and conceptual integration of psychological frameworks to describe MDMA’s potential therapeutic effects.
D) **RAP interviews:** To assess changes in reflective functioning, each RAP interview will be scored using procedures in the Reflective Functioning Manual [27]. Changes in relational patterns will be examined using the CCRT paradigm [24]. We hypothesise that MDMA may predict changes in CCRT (particularly the *wish* component), with reflective functioning acting as a moderator. Interview order blinding, independent coding, and periodic calibration will be used to enhance coding accuracy.

#### 2.6.2 Questionnaires.

Given the small sample size and absence of a control group, questionnaire responses will be analysed descriptively (e.g., measures of central tendency and variation). Any inferential analyses will be exploratory and interpreted cautiously, as the study is not designed to test hypotheses regarding the measured psychological constructs.

#### 2.6.3 Journaling.

Journal data will be analysed using thematic analysis, with the aim of enriching the developing model of MDMA’s subjective mechanisms. Exploratory quantitative analysis (e.g., valence-based indices) may also be undertaken.

#### 2.6.4 Data synthesis.

Following completion of the analyses above, the research team will develop a model of MDMA’s therapeutic mechanisms. Findings from qualitative interviews will be compared across individuals to identify convergence and divergence and to generate hypotheses about factors contributing to these patterns. This work will be informed by the ‘constant comparison’ technique, which supports development of “*conceptual frameworks or theories through building inductive analysis from the data”* [16] (p. 187). Integrating questionnaires, journaling, and RAP-derived indices will support hypothesis generation linking specific subjective experiences to generalisable psychological processes. This synthesis will culminate in a dynamic model of mechanisms-in-action intended to be amenable to testing in future experimental settings. Table 1 summarises candidate psychological constructs which may be included in the model and data collection techniques through which these will be elicited.

### 2.7 Patient and public involvement

Stakeholder consultations with four psychotherapists (representative of the target sample) were used to refine the data-collection sequence, interview probes, data collection tools, and recruitment strategy. Overall feedback indicated good acceptability of the protocol. Suggested changes were minor and implemented via amendments approved by the UCL Research Ethics Committee. Key changes included:

- Addition of recruitment avenues (specialist conferences and professional networks).
- Specification of contingency plans if recruitment proved difficult (e.g., removing a seniority criterion from eligibility criteria). MDMA-AP.
- Rewording and addition of interview prompts.
- Rewording of the Participant Information Leaflet to emphasise flexibility in scheduling experimental sessions.

Recognising the potential risk of adverse residual (post-acute) effects of MDMA on participants’ work schedules and clinical commitments at higher doses, we decided to limit the maximum per-session dose to 120 mg. We also decided to have a relatively short minimum interval between dosing sessions (one week) as this was deemed to provide greater flexibility to participants.

### 2.8 Safety considerations

Safety will be ensured through screening, on-site health assessment, on-site physician or research nurse oversight, physiological monitoring, and adverse-event protocols. We will use conservative eligibility criteria to minimise the likelihood of adverse cardiac effects. Eligibility will be evaluated through two stages of screening (online and telephone) and an on-site health assessment in Session 1, including 6-lead EKG and BP assessment, to confirm medical suitability for MDMA administration. Screening results, EKG, and BP readings will be reviewed by a medical doctor or research nurse prior to Session 2, at which point EKG and BP will be re-assessed. Abnormalities detected in Session 1 will lead to exclusion; participants will be informed of the reason for exclusion and advised to monitor BP and/or consult their family doctor as appropriate. A medical doctor or research nurse will supervise drug administration and will be present during the initial 120 min post-administration to monitor physiological responses. Each dosing session will be facilitated by up to two trained researchers, or by a trained researcher and trainee under the supervision of an experienced researcher.

We plan to perform assessments of participants’ physiological responses at pre-specified intervals: baseline (pre-dose), t = 30, 45, and 60 min, then at 30-min intervals until t = 180 min, after which monitoring will be hourly. If this interferes with the participants’ immersion in the experience, the number of assessments may be reduced (although baseline, peak and end-point assessments will be retained). Participants will remain under direct observation for the full duration of each dosing session. Adverse events that occur during the study period (from the first dosing session to the last data collection session) will be systematically reported. Any researcher involved in direct participant care will be trained by a senior researcher to identify and document adverse events, with actions taken and resolutions recorded and reported to the ethics committee as required. Our protocol specifies clear thresholds for intervention (e.g., when the PI and/or supervising medic should intervene; when the dosing session should be aborted; when emergency services should be enlisted).

At least one researcher with first aid training will be present or immediately available. Any serious adverse events will be promptly reported to the study medic (who will remain on call after the initial 120-min monitoring period) and to the Principal Investigator, who will also be on call (if not present) during dosing sessions to coordinate responses to immediate needs or safety concerns. Staff in the psychopharmacology unit have substantial experience in supporting participants under altered states of consciousness, including in studies of DMT, psilocybin, ketamine, and nitrous oxide.

We have also considered the possible adverse effects of participation in interviews and questionnaires. Interview procedures are unlikely to be distressing for this participant group, as they elicit beliefs and accounts of experiences expected to be familiar in professional practice (e.g., relationship with self, aspects of the self, and relational experiences). Psychodynamic psychotherapists routinely engage in self-reflection and discuss relationship patterns in supervision. In addition, their training typically involves long-term personal psychotherapy, which is likely to support self-awareness and resilience in exploring internal experience. Participants are therefore not expected to experience the interviews as unfamiliar or intrusive. In the RAP, participants will be able to identify target relational episodes independently and they will not be asked to disclose any identifiable details of those involved. Importantly, participants will not be asked to discuss sensitive topics or events or trauma content while under the influence of MDMA, and the interviewers will redirect to present-moment phenomenology if sensitive narratives emerge at that point.

Despite these protections, participants will be provided with a mental health resource leaflet listing available supports should they require them after participation. Researchers will remain alert to signs of distress and will monitor participants throughout the study, offering support, advice, and signposting as needed.

### 2.9 Data management plan

Data will be managed internally by the research team. A data monitoring committee is not required given that the study is not a clinical trial. Nonetheless, confidentiality is an overriding requirement and will be maintained according to rigorous institutional and national standards.

Personally identifiable data will be collected during screening and stored on a secure institutional facility. These data will be destroyed when data collection is complete. Signed informed consent forms will be retained in a secure University-provided facility for three years after study completion. Pseudonymised interview transcripts, questionnaire data, and journal entries will be stored on access-controlled storage for three years following study completion. After this point, pseudonymised identifiers will be removed, and non-identifiable data will be retained indefinitely for study reporting and publication.

Since the target sample belongs to a small local professional community, particular attention will be devoted to removing sections of the interviews that may indirectly identify participants from published interview quotes. Only researchers directly involved in the study and with approved General Data Protection Regulation (GDPR) training will have access to original data. Other researchers may be granted access in the future to data that can be fully anonymised.

### 2.10 Timeline

The study has not generated results. Recruitment is expected to begin in the second quarter of 2026 and to be completed by the second or third quarter of 2027, with results available in 2028.

## 3. Discussion

This protocol outlines a qualitative mechanistic study of the entactogen MDMA, which has shown promise in the treatment of PTSD. By combining phenomenological and thematic interviewing, RAP interviews with CCRT and reflective functioning scoring, longitudinal journaling, and psychological questionnaires, the study is designed to support a fine-grained mapping of experiential processes across acute, sub-acute, and integration phases. By purposively sampling trained psychodynamic psychotherapists and leveraging their clinical expertise and self-reflective capacities, we aim to interrogate psychological mechanisms that have been under-researched in neurobiologically focused studies, including changes in self-related processing, interpersonal patterns, reflective functioning, and meaning-making. This use of the first-person accounts of expert clinician-participants as high-fidelity reports for probing MDMA-related internal and relational phenomena is a distinctive feature of the protocol. The intended outcome is a theoretically grounded, testable model of MDMA’s psychological mechanisms of action, providing a bridge between first-person experience and constructs amenable to operationalisation in future clinical and experimental trials. We suggest that this methodological orientation adds value to the current research landscape by enabling a nuanced examination of psychological and interpersonal processes that are difficult to capture using standard quantitative and neuroscientific paradigms.

Several features strengthen the study design. First, sequential, time-anchored interviews during dosing sessions prioritise experience-near description, while the subsequent IPA interview explicitly invites longer-term meaning-making. In this way, both immediate effects and the products of longer-term integration are captured. Second, triangulation across methods (phenomenological interviews, RAP interviews, narrative journals, and descriptive psychometrics) is intended to generate testable hypotheses and strengthen analytic validity. Third, focusing on psychodynamic clinicians maximises the sample’s *information* *power* [20]: participants’ training in introspection and in communicating complex psychological processes increases the likelihood of detailed, nuanced accounts, thereby enhancing the efficiency and depth of qualitative inference with a modest sample size.

The study also has limitations. The open-label, non-controlled design means that expectancy and demand effects are likely to shape participants’ subjective experiences. Participants’ professional familiarity with psychotherapy processes and their theoretical commitments could both deepen insight into how MDMA achieves its effects and, conversely, constrain interpretation by privileging familiar explanatory frames over novel accounts. Our data is self-reported and is therefore vulnerable to memory and social desirability biases, despite efforts to capture in-the-moment experience. We seek to mitigate these limitations through: (i) phase-specific, idiographic analysis of interviews prior to cross-phase synthesis (2.6.1.A-C); (ii) triangulation across analysts at key stages (2.6.4); (iii) explicit attention to convergence and divergence during constant-comparison synthesis (2.6.4); and (iv) integration of experiential themes with structured indices, including CCRT and reflective functioning ratings (2.6.1.D).

An additional limitation is that our participants will not be encouraged to engage with spontaneously emerging traumatic materials during the dosing sessions. Given that PTSD is the primary indication for MDMA-AP, this may limit the insights we can gain about changes in traumatic memories processing. However, the team considered it ethically problematic to encourage participants to engage with and discuss traumatic materials when they might not, ordinarily, be inclined to do so (i.e., outside of the MDMA state). Equally, avoiding trauma processing will enable us to preserve the interpretability of mechanisms because we will not be conflating drug effects with exposure-based therapeutic processes.

Despite these constraints, we expect the study to contribute substantively to our understanding of MDMA as a therapeutic agent. A qualitative, mechanism-focused account of MDMA’s action may clarify which elements of adjunctive psychotherapy are necessary, for whom, and when, thereby supporting optimisation of psychotherapy principles and protocols. Candidate mechanisms – such as shifts in relational patterns and self–other representations, their hypothesised role, temporal ordering, and contextual conditions – can be formalised into hypotheses for experimental and clinical testing.

Beyond MDMA, the protocol offers a transferable template for qualitative mechanistic examination of other psychedelic compounds, promoting the development of experience-led, evidence-based models of psychedelic-assisted therapies grounded in first-person phenomenology. Moreover, the primary output of this project – a theory (or theories) of psychological change induced by MDMA – may have implications beyond therapeutic refinement. For example, it may generate constructs and hypotheses that may inform the development of novel quantitative tools in behavioural psychopharmacology that draw on psychoanalytic concepts likely to be affected by MDMA (e.g., questionnaires or behavioural tasks assessing loosening of defences and access to previously repressed or suppressed material; *‘corrective emotional experiences’*).

Substantive amendments to the protocol will be reported transparently in subsequent publications. Such changes will be data-driven, agreed within the team, and submitted to the Research Ethics Committee for approval. Any pattern of adverse physiological or psychological events exceeding pre-specified thresholds (per our pre-specified procedure) will result in study interruption. In the event of early termination, we will document reasons and analyse accrued data with appropriate caveats. Dissemination plans include conference presentations and publication in peer-reviewed academic journals.

## References

[1] Wolfgang AS, Fonzo GA, Gray JC, Krystal JH, Grzenda A, Widge AS, et al. MDMA and MDMA-assisted therapy. Am J Psychiatry. 2025;182(1):79–103. doi: 10.1176/appi.ajp.20230681 39741438

[2] Yehuda R, Lehrner A. Psychedelic therapy-a new paradigm of care for mental health. JAMA. 2023;330(9):813–4. doi: 10.1001/jama.2023.12900 37651148

[3] Mitchell JM, Bogenschutz M, Lilienstein A, Harrison C, Kleiman S, Parker-Guilbert K, et al. MDMA-assisted therapy for severe PTSD: a randomized, double-blind, placebo-controlled phase 3 study. Nat Med. 2021;27(6):1025–33. doi: 10.1038/s41591-021-01336-3 33972795 PMC8205851

[4] Mitchell JM, Ot’alora G M, van der Kolk B, Shannon S, Bogenschutz M, Gelfand Y, et al. MDMA-assisted therapy for moderate to severe PTSD: a randomized, placebo-controlled phase 3 trial. Nat Med. 2023;29(10):2473–80. doi: 10.1038/s41591-023-02565-4 37709999 PMC10579091

[5] Goodwin GM, Malievskaia E, Fonzo GA, Nemeroff CB. Must psilocybin always “assist psychotherapy”? Am J Psychiatry. 2024;181(1):20–5. doi: 10.1176/appi.ajp.20221043 37434509

[6] Zamaria JA, Fernandes-Osterhold G, Shedler J, Yehuda R. Psychedelics assisting therapy, or therapy assisting psychedelics? The importance of psychotherapy in psychedelic-assisted therapy. Front Psychol. 2025;16:1505894. doi: 10.3389/fpsyg.2025.1505894 39973948 PMC11835864

[7] Yehuda R, Lehrner A, Sperka M, Glatman Zaretsky T, Pratchett LC, Haznedar MM, et al. The case for an integrative model: hypotheses and rationale for integrative MDMA-Assisted Psychotherapy (IMAP). Psychedelics. 2026;3:100008. doi: 10.1016/j.psyche.2026.100008

[8] Mithoefer A, Jerome L, Ruse J, Doblin R, Gibson E, Marcela Ot’alora G. A manual for MDMA-assisted psychotherapy in the treatment of posttraumatic stress disorder. Santa Cruz (CA): MAPS; 2013.

[9] Levine PA. In an unspoken voice: how the body releases trauma and restores goodness. North Atlantic Books; 2010.

[10] Schwartz RC. Introduction to the internal family systems model. Trailheads Publications; 2001.

[11] Morland LA, Knopp K, Khalifian C, Stauffer CS. Charting a new frontier: integrating cognitive behavioral therapy approaches with 3, 4-methylenedioxymethamphetamine. Cogn Behav Pract. 2025;32(3):315–20.

[12] Luoma JB, Lear MK, Yi K, Pilecki B. Utilizing in vivo and imaginal exposure in the context of MDMA-assisted therapy for social anxiety disorder: a case report. Cogn Behav Pract. 2025;32(3):351–63.

[13] Yaden DB, Earp D, Graziosi M, Friedman-Wheeler D, Luoma JB, Johnson MW. Psychedelics and psychotherapy: cognitive-behavioral approaches as default. Front Psychol. 2022;13:873279. doi: 10.3389/fpsyg.2022.873279 35677124 PMC9169963

[14] Breeksema JJ, Niemeijer AR, Krediet E, Vermetten E, Schoevers RA. Psychedelic treatments for psychiatric disorders: a systematic review and thematic synthesis of patient experiences in qualitative studies. CNS Drugs. 2020;34(9):925–46. doi: 10.1007/s40263-020-00748-y 32803732 PMC7447679

[15] Robison R, Barrow R, Conant C, Foster E, Freedman JM, Jacobsen PL, et al. Single treatment with MM120 (lysergide) in generalized anxiety disorder: a randomized clinical trial. JAMA. 2025;334(15):1358–72. doi: 10.1001/jama.2025.13481 40906494 PMC12412041

[16] Charmaz K. Constructing grounded theory: a practical guide through qualitative analysis. Sage; 2006.

[17] Gelso CJ, Hill CE, Kivlighan DM Jr. Transference, insight, and the counselor’s intentions during a counseling hour. J Couns Dev. 1991;69(5):428–33.

[18] Liester MB, Grob CS, Bravo GL, Walsh RN. Phenomenology and sequelae of 3,4-methylenedioxymethamphetamine use. J Nerv Ment Dis. 1992;180(6):345–52; discussion 353-4. doi: 10.1097/00005053-199206000-00001 1350613

[19] Bateman AW, Fonagy P. Handbook of mentalizing in mental health practice. American Psychiatric Pub; 2019.

[20] Malterud K, Siersma VD, Guassora AD. Sample size in qualitative interview studies: guided by information power. Qual Health Res. 2016;26(13):1753–60. doi: 10.1177/1049732315617444 26613970

[21] Hennink MM, Kaiser BN, Marconi VC. Code saturation versus meaning saturation: how many interviews are enough? Qual Health Res. 2017;27(4):591–608. doi: 10.1177/1049732316665344 27670770 PMC9359070

[22] Smith JA, Larkin M, Flowers P. Interpretative phenomenological analysis: theory, method and research. London: Sage; 2021.

[23] Petitmengin C. Micro-phenomenology as coming into contact with experience: subtilisation, surprises, and liberation. In: Practicing embodied thinking in research and learning. Routledge; 2024. p. 119–28.

[24] Luborsky L, Crits-Christoph P. Understanding transference: the core conflictual relationship theme method. American Psychological Association; 1998.

[25] Egozi S, Talia A, Wiseman H, Tishby O. The experience of closeness and distance in the therapeutic relationship of patients with different attachment classifications: an exploration of prototypical cases. Front Psychiatry. 2023;14:1029783. doi: 10.3389/fpsyt.2023.1029783 37398585 PMC10311418

[26] Wiseman H, Tishby O. Applying relationship anecdotes paradigm interviews to study client-therapist relationship narratives: core conflictual relationship theme analyses. Psychother Res. 2017;27(3):283–99. doi: 10.1080/10503307.2016.1271958 28102111

[27] Fonagy P, Target M, Steele H, Steele M. Reflective-functioning manual version 5 for application to adult attachment interviews. 1998.

[28] Gu J, Baer R, Cavanagh K, Kuyken W, Strauss C. Development and psychometric properties of the Sussex-Oxford compassion scales (SOCS). Assessment. 2020;27(1):3–20. doi: 10.1177/1073191119860911 31353931 PMC6906538

[29] Spreng RN, McKinnon MC, Mar RA, Levine B. The Toronto Empathy Questionnaire: scale development and initial validation of a factor-analytic solution to multiple empathy measures. J Pers Assess. 2009;91(1):62–71. 19085285 10.1080/00223890802484381 PMC2775495

[30] Campbell C, Tanzer M, Saunders R, Booker T, Allison E, Li E, et al. Development and validation of a self-report measure of epistemic trust. PLoS One. 2021;16(4):e0250264. doi: 10.1371/journal.pone.0250264 33861805 PMC8051785

[31] Fraley RC, Heffernan ME, Vicary AM, Brumbaugh CC. The experiences in close relationships-relationship structures questionnaire: a method for assessing attachment orientations across relationships. Psychol Assess. 2011;23(3):615–25. doi: 10.1037/a0022898 21443364

[32] Barkham M, Hardy GE, Startup M. The IIP-32: a short version of the Inventory of Interpersonal Problems. Br J Clin Psychol. 1996;35(1):21–35. doi: 10.1111/j.2044-8260.1996.tb01159.x 8673033

[33] Watson D, Clark LA, Tellegen A. Development and validation of brief measures of positive and negative affect: the PANAS scales. J Pers Soc Psychol. 1988;54(6):1063–70. doi: 10.1037//0022-3514.54.6.1063 3397865

[34] Morean ME, de Wit H, King AC, Sofuoglu M, Rueger SY, O’Malley SS. The drug effects questionnaire: psychometric support across three drug types. Psychopharmacology (Berl). 2013;227(1):177–92. doi: 10.1007/s00213-012-2954-z 23271193 PMC3624068

[35] Roseman L, Haijen E, Idialu-Ikato K, Kaelen M, Watts R, Carhart-Harris R. Emotional breakthrough and psychedelics: validation of the emotional breakthrough inventory. J Psychopharmacol. 2019;33(9):1076–87. doi: 10.1177/0269881119855974 31294673

[36] Studerus E, Gamma A, Vollenweider FX. Psychometric evaluation of the altered states of consciousness rating scale (OAV). PLoS One. 2010;5(8):e12412. doi: 10.1371/journal.pone.0012412 20824211 PMC2930851

[37] Strickland JC, Garcia-Romeu A, Johnson MW. The mystical experience questionnaire 4-item and challenging experience questionnaire 7-item. Psychedelic Med (New Rochelle). 2024;2(1):33–43. doi: 10.1089/psymed.2023.0046 40051759 PMC11658653

[38] Zentner M, Grandjean D, Scherer KR. Emotions evoked by the sound of music: characterization, classification, and measurement. Emotion. 2008;8(4):494–521. doi: 10.1037/1528-3542.8.4.494 18729581

[39] Braun V, Clarke V. One size fits all? What counts as quality practice in (reflexive) thematic analysis? Qual Res Psychol. 2021;18(3):328–52.

