# Supplementary material for: Protocol for a qualitative mechanistic study of MDMA with a sample of psychoanalytic psychotherapists: A phenomenological investigation
Source: PLoS One. 2026 Jun 18;21(6):e0350728. doi: 10.1371/journal.pone.0350728 (PMC13278426; doi:10.1371/journal.pone.0350728)
Supplement: Attachment — Submitted filename: Grob PsychiatristsMDMA.pdf [file pone.0350728.s002.pdf]

UC Irvine
UC Irvine Previously Published Works

Title
Phenomenology and Sequelae of 3,4-Methylenedioxymethamphetamine Use

Permalink
https://escholarship.org/uc/item/4z26h1n9

Journal
The Journal of Nervous and Mental Disease, 180(6)

ISSN
0022-3018

Authors
LIESTER, MITCHELL B
GROB, CHARLES S
BRAVO, GARY L
et al.

Publication Date
1992-06-01

DOI
10.1097/00005053-199206000-00001

Copyright Information
This work is made available under the terms of a Creative Commons Attribution License, available at https://creativecommons.org/licenses/by/4.0/

Peer reviewed

eScholarship.org Powered by the California Digital Library
University of California

THE JOURNAL OF

NERVOUS AND MENTAL DISEASE

VOL. 180, NO. 6
June 1992
SERIAL NO. 1315

Phenomenology and Sequelae of 3,4-Methylenedioxymethamphetamine Use

MITCHELL B. LIESTER, M.D., CHARLES S. GROB, M.D., GARY L. BRAVO, M.D., AND ROGER N. WALSH, M.D., PH.D.[1]

3,4-Methylenedioxymethamphetamine (MDMA) has been at the center of a debate over its potential benefits as an adjunct to psychotherapy versus its capability for neurotoxic effects and is currently classified as a Schedule 1 drug by the Drug Enforcement Administration (DEA). However, as yet, there is very little methodological data on the subjective experience of the MDMA-induced state or its psychological and behavioral sequelae. The present study was, therefore, designed to obtain this kind of information. Twenty psychiatrists who had taken MDMA previously were evaluated using a semistructured interview. Subjective experience of the actual MDMA-induced state, as well as both short-term (<1 week) and relatively longer term (>1 week) sequelae, were examined retrospectively. Side effects, insight gained, pleasure, and intensity of the MDMA experience were evaluated as were the influence of set and setting at the time the MDMA was taken and the dosage utilized. Finally, the authors discuss methodological problems and limitations of a study of this type.

—*J Nerv Ment Dis* 180:345–352, 1992

3, 4-Methylenedioxymethamphetamine (MDMA) is a methoxylated amphetamine that is chemically related to both hallucinogens and stimulants. Although first synthesized and patented in Europe in 1914 (Downing, 1986), MDMA remained in relative obscurity until the late 1960s, when it reappeared in the western United States (Seymour, 1986). During the 1970s and early 1980s, MDMA gained popularity as both an adjunct to psychotherapy (Downing, 1986; Greer and Tolbert, 1986; Greer and Strassman, 1985) and as a recreational drug (Peroutka, 1987). The popularity of the drug may be related to its reported ability to increase self-confidence and self-acceptance, lower defenses, and induce feelings of empathy and love (Grinspoon and Bakalar, 1986). MDMA's recent utilization as an adjunct to psychotherapy has historical antecedents in the 1950s and early 1960s, with efforts designed to explore both hallucinogen-assisted (Grinspoon and Bakalar, 1979) and amphetamine-assisted (Pohlman, 1957) psychotherapies.

Reports of neurotoxicity in laboratory animals from a related compound, methylenedioxyamphetamine (MDA; Ricuarte et al., 1985), however, led the Drug Enforcement Administration (DEA) to place MDMA into Schedule 1 in July of 1985. Schedule 1 is reserved for drugs that are deemed to have a high potential for abuse, have no currently accepted medical use in the United States, and lack accepted safety for use under medical supervision. Controversy over this classification led to subsequent court hearings and the recommendation of the administrative judge that, as there was indeed a valid argument for an accepted medical use of MDMA, it be removed from Schedule 1 and placed in the less restrictive Schedule 3 (Barnes, 1988; Lawn 1988a). This reclassification was temporary, however, because DEA officials overturned the court decision and MDMA was once again assigned to Schedule 1 in March of 1988 (Lawn, 1988b).

Concerns have been raised that MDMA may induce short-term as well as long-term adverse effects, some of severe and perhaps life-threatening proportions. Side effects include effects similar to those of amphetamines (*e.g.*, tachycardia, dry mouth, palpitations, trismus, bruxism, nausea, and insomnia [Greer and Tolbert, 1986; Peroutka et al., 1988]), impaired judgment and gait (Downing, 1986), panic attacks (Whitaker-Azmitia and Aronson, 1989), and human death (Dowling et al., 1987). Neurotoxicity has been reported in laboratory animals (Commins et al., 1987; Schmidt, 1987; Schmidt and Taylor, 1987), including axonal changes in seroton-

[1]Department of Psychiatry, University of California, Irvine Medical Center, 101 City Drive, Route 88, Orange, California 92668. Send reprint requests to Dr. Grob.

ergic neurons of nonhuman primates given relatively low dosages of MDMA (Ricaurte et al., 1988).

Examination of many of these studies, however, raises several questions. In Dowling et al.'s (1987) report of five deaths in which MDMA was detected in blood by postmortem toxicology, independent and potentially lethal medical factors were present in each instance. These included atherosclerotic cardiovascular disease, idiopathic cardiomyopathy, bronchial asthma, electrocution, and toxicology screens positive for barbiturates, narcotic analgesics, and alcohol. Reports of neurotoxicity are complicated by differences in the effects of MDMA on different species of laboratory animals (Battaglia et al., 1988; Logan et al., 1988; Stone et al., 1987) and by the fact that long-term studies suggest that MDMA-induced changes in serotonin neurons may be only temporary (Battaglia et al., 1988). Studies designed to assess serotonin function in humans with a history of heavy recreational MDMA use (Price et al., 1989) have on careful review been found to be methodologically flawed (Grob et al., 1990). The clinical implications of a depletion in serotonin neuronal terminals remain unclear, inasmuch as there have been no documented clinical cases of MDMA-induced serotonergic neurotoxicity; *e.g.*, there have been no reports of expected mood, sleep, appetite, aggressive, or sexual dysregulation. Furthermore, a widely prescribed and approved anorectic medication, fenfluramine, has a significantly greater degree of neurotoxicity on the serotonergic neurotransmitter system (Schuster et al., 1986), and yet has not been associated with any adverse clinical sequelae, remains classified as a Schedule 4 drug, and continues to be marketed (Derome-Tremblay and Nathan, 1989). Reports of panic attacks have left unanswered questions about whether these episodes met DSM-III-R criteria for panic disorder or were transient episodes of anxiety.

Despite all this clinical, pharmacological, and legal attention, few studies have examined the phenomenology of the MDMA-induced state, behavioral and psychological sequelae, or the beneficial effects that users claim for it. In one of the few studies that did investigate possible beneficial effects of MDMA, Greer and Tolbert (1986) reported on the experiences of 29 subjects. The subjects studied claimed enhanced communication (100%), increased feelings of intimacy (93%), cognitive benefit (*e.g.*, an expanded mental perspective, insight into personal patterns or problems, and improved self-examination skills, etc.; 76%), euphoria or loving feelings (55%), greater self-confidence or self-acceptance (34%), lowered defenses (34%), and transcendent experiences (17%). Other studies have also included reports of a heightened sense of closeness with other people (Peroutka et al., 1988) and heightened sensual awareness (Downing, 1986).

The questions raised by this study are in many ways similar to those faced by researchers in the 1960s who investigated hallucinogens (*e.g.*, LSD). A review of such studies reveals a disparate array of conflicting perspectives. Findings ranged from the demonstration of a strong association between the frequent use of hallucinogens and severe underlying psychopathology (Barron et al., 1970; Blacker et al., 1968; Smart and Jones, 1970; Welpton, 1968) to the assessment that even limited exposure to these potent mind-altering substances could eventuate in profound, positive, and long-lasting changes in underlying personality structure (Sherwood et al., 1962; Unger, 1963). Until research into the clinical effects and therapeutic potential of hallucinogens was abruptly terminated because of public health and medical concerns as well as social and political pressures, numerous studies were conducted to answer these vexing questions. Significant among them were McGlothlin et al.'s (1967) observation that although more than half of their normal subjects receiving experimental LSD subjectively reported lasting positive effects, it was not possible to corroborate such findings through objective psychological measurement. Other investigators (Savage et al., 1964, 1966), however, reported significant positive effects at 1 and 2-year follow-up assessments of long-term psychological change following only one high dose hallucinogen session. Unfortunately, the societal and political turmoil experienced during this historical period as well as highly publicized public health and medical concerns prevented further examination of these confounding research questions. The present study, although by necessity retrospective in nature, is a preliminary attempt designed to clarify some of these unanswered questions.

The present study retrospectively investigated the subjective reports of the phenomenology and the psychological and behavioral sequelae of MDMA use by 20 psychiatrists. The authors also attempt to more clearly define some of the previously reported effects of the drug.

Methods

Twenty subjects volunteered for participation in this study. All participants met the inclusion criteria of being doctors of medicine, having a minimum of at least 1 year's training in a psychiatric residency program, and having previously used MDMA. No subject who met these criteria was excluded. We selected this sample for study because we believed that psychiatrists would be knowledgeable of psychological phenomena and trained to observe and analyze their own internal experiences. Also, it presented an opportunity to ask physicians who had personal experience with the drug about their opinions regarding its therapeutic and abuse potentials.

After explaining the study, obtaining informed consent, and assuring confidentiality, one interviewer (M. B. L.) conducted a semi-structured interview. The interview included questions about demographic information, medical and psychiatric history, frequency of MDMA use, dose, set, and setting. Psychological and physiological effects, including adverse effects and sequelae to the experience, were explored, as were changes in the effects of MDMA with repeated use. Similarities between the effects of MDMA and other previously used psychoactive substances were also examined.

Subjects were queried about their attitudes toward MDMA, including concerns about reported neurotoxic effects, opinions regarding abuse and therapeutic potentials, and the religious or spiritual significance of their MDMA experiences. Finally, each subject was asked whether he or she had ever given MDMA to a patient as an adjunct to psychotherapy.

Results

Demographic Information

The 20 subjects consisted of 18 men and two women ranging in age from 28 to 55 years (mean ± SD, 35.9 ± 7.2 years). All subjects resided in Southern California and were recruited over several months through word of mouth. The subjects had been in their psychiatric careers from 1 to 25 years (7 ± 6.5 years). Fifty-five percent (11 of 20) had completed their psychiatric residency, while 45% (9 of 20) were presently in a psychiatric training program. All subjects had been in psychotherapy at some point in their lives, with the length of treatment ranging from 5 months to 10 years. Thirty percent (6 of 20) reported ongoing medical problems. Five of these subjects were taking prescription medications for their conditions (*i.e.*, migraine headaches, hypertension, glaucoma, ulcerative colitis, and arthritis) and a sixth described chronic low back pain but was receiving no medical treatment. Whereas this was clearly not a drug-naive sample, since all subjects had a history of drug experimentation, most commonly with marijuana, none of the subjects had a history suggestive of psychoactive substance abuse or psychoactive substance dependence as defined by DSM-III-R. Fifty-five percent (11 of 20) reported having a regular "spiritual" or meditative practice.

MDMA Use

The number of times subjects had previously ingested MDMA ranged from one to 25 (4.2 ± 5.1). Six (30%) of the subjects were able to recall the dose of MDMA they had taken. Among these subjects, the reported dose range was 100 to 200 mg (147 ± 28 mg). A second dose was utilized on at least one occasion by 70% (14 of 20) of subjects with a dose range of 30 to 125 mg (75 ± 33 mg). The length of time between the last use of MDMA and the interview ranged from 1 day to 4 years (400 ± 351 days; median time, 360 days).

Set and Setting

A majority of the subjects (80% [16 of 20]) prepared themselves in some way for their MDMA experience. Common preparations included speaking with others who had had experience with MDMA, fasting, meditating, selecting a quiet setting where disturbances were unlikely, and being with close friends when the MDMA was taken. All subjects had some knowledge of the effects of MDMA before taking it and 65% (13 of 20) reported having positive expectations about their experience. Only 35% (7 of 20) of subjects recalled having been given instructions on how to use MDMA.

When subjects were asked about their intention or purpose for using MDMA, 80% (16 of 20) listed self-exploration or personal growth, 30% (6 of 20) stated they utilized the drug for enhancing interpersonal relationships, 25% (5 of 20) desired a pleasant experience, and 20% (4 of 20) mentioned curiosity (subjects often gave more than one response to this question). Twenty-five percent (5 of 20) of subjects reported taking other psychoactive substances with MDMA on at least one occasion. The most common were marijuana (15% [3 of 20]), alcohol (15% [3 of 20]), and benzodiazepines (5% [1 of 20]).

Eighty-five percent (17 of 20) of subjects regulated the environment to enhance their MDMA experience. The most frequently reported methods were listening to music or poetry, selecting a natural setting (*e.g.*, a forest, beach, etc.), and utilizing a blindfold (to decrease visual input). Of the 17 subjects who regulated the environment, 14 of 17 (82%) reported that the effect of the environment on them was enhanced during the MDMA experience, two subjects reported no change, and one subject felt the environment was "more meaningless." Examples of the enhanced effect of the environment on the subjects can be found in the following quotations: "the music had much more depth, richness and clarity"; "the music seemed clearer . . . I could hear things I hadn't heard before even though I'd heard the song before . . . I was more attentive to the words . . . the notes were very clear and stood out"; "colors were brighter . . . there was a sensory perceptual enhancement"; "I was much more in harmony with nature. I could feel the power of being in nature"; and "the poetry and the music were more meaningful. My receptivity to their emotional impact was much greater."

Subjective Effects of MDMA

The phenomenological experiences reported most often by subjects are listed in Table 1. Experiences

TABLE 1
Phenomenology of MDMA

	%
Altered time perception	90
Increased ability to interact with or be open with others	85
Decreased defensiveness	80
Decreased fear	65
Decreased sense of separation or alienation from others	60
Changes in visual perception	55
Increased awareness of emotions	50
Decreased aggression	50
Speech changes	45
Aware of previously unconscious memories	40
Decreased obsessiveness	40
Cognitive changes	40
Decreased restlessness/agitation	30
Decreased impulsivity	25
Decreased compulsiveness	20
Decreased anxiety	15
Altered perception of spatial relationships	15
Decreased desire for sleep	10
Increased libido	10

reported most frequently included: altered perception of the passage of time (90% [18 of 20]), increased ability to interact with or be open with others (85% [17 of 20]), decreased defensiveness (80% [16 of 20]), decreased fear (65% [13 of 20]), decreased sense of separation or alienation from others (*i.e.*, changes in ego boundaries; 60% [12 of 20]), changes in visual perception (55% [11 of 20]), increased awareness of emotions (50% [10 of 20]), decreased feelings of aggression (50% [10 of 20]), alterations in speech patterns (45% [9 of 20]), awareness of previously unconscious memories (40% [8 of 20]), decreased obsessiveness (40% [8 of 20]), and cognitive changes (40% [8 of 20]). The phenomenological nature of these experiences can be elucidated by direct quotations from subjects.

Reports of altered time perception were highly variable. Examples included reports of time being "compressed," "dilated," "expanded," "slowed down," and "sped up." One subject experienced time both speeding up and slowing down in the same MDMA experience. Others were aware that their perception of time was in some way altered, but were unable to describe the nature of the alteration.

Of the 17 subjects who reported an increased openness or ability to interact with others, two separate subjects stated that they each became engaged within 1 month after taking MDMA. One subject related that this occurred because "we saw other reasons for doing things which we'd thought we were doing because we didn't care for each other. We saw the love underneath it all." The other subject stated "we focused on how we were defensive with each other." Follow-up 2 years later revealed that both subjects had married and that each couple had remained married.

Subjects generally reported that they were less aware of their own boundaries and described experiencing less distinction between their "self" and "others." None of the subjects reported that ego boundary changes caused problems during or after their MDMA experiences. Several reported an enhancement of their personal relationships and professional performance as a result of these changes. Quotations included the following: "I had a sense of being more connected and less separate . . . I felt more unity with people . . . I had less idea of them being separate"; "(I felt) more united with the world and other people"; and "my ego boundaries become porous . . . there is a sharing of ego boundaries with the persons you're with."

Cognitive changes involved shifts in the form and content of cognition. Characteristic comments included the following: "slowed thoughts"; "mental slowing"; "increased tendency to think about relationships"; "strong shift from the mundane toward oneness, spiritualness . . . shift to a more positive cognitive set"; "called a friend in Florida, but I was confused. I thought it was 3 hours earlier than it was"; and "unconventional thoughts . . . new ideas . . . reframed."

Visual changes generally involved an intensification of visual perception: "colors were more intense"; "perceptual clarity"; "heightened awareness . . . more intense"; "enhancement of color and light . . . I perceived more detail"; and "a perceptual accentuation of what I was focusing on." Four of the subjects, however, reported a change in the content of visual perception. One reported experiencing visual images of squares and colors during an MDMA session in which a blindfold was worn. A second subject reported seeing "patterns, designs, and colors." These were present only when the subject's eyes were closed and when MDMA was taken in combination with marijuana. A third subject saw "patterns of dots" and experienced a visual illusion (*i.e.*, " the walls moved") when MDMA was combined with alcohol. A fourth subject reported changes in visual perception during an MDMA session at night in a room where no lights were turned on. Thus, among the four subjects who experienced visual imagery, illusions, or hallucinations, perceptual changes occurred only when there was either an environmentally induced reduction in visual input (*i.e.*, a blindfold or darkened room) or when another psychoactive substance was combined with MDMA (*i.e.*, alcohol or marijuana).

Adverse Effects

It should be noted that the decision to label effects as adverse was made by the investigators and not the subjects. In some instances, subjects spontaneously reported no adverse or unpleasant responses to these effects of the drug (*e.g.*, decreased appetite). The most

common adverse effects that occurred during the MDMA-induced state are listed in Table 2 and include decreased desire to perform mental or physical tasks (70% [14 of 20]), decreased appetite (65% [13 of 20]), trismus (*i.e.*, jaw clenching; 50% [10 of 20]), decreased libido (45% [9 of 20]), increased restlessness and agitation (35% [7 of 20]), bruxism (*i.e.*, grinding of the teeth; 30% [6 of 20]), increased anxiety (25% [5 of 20], although in one case the anxiety was related to the sudden appearance of a dog and disappeared when the dog left), and decreased ability to perform mental or physical tasks (20% [4 of 20]). Other experiences that were not specifically asked about but were mentioned during the interviews included nystagmus (10% [2 of 20]), diaphoresis (5% [1 of 20]), tactile illusion ("a blanket felt like the wind"; 5% [1 of 20]) and an intensified feeling that life is meaningless (5% [1 of 20]). There were no reports of auditory hallucinations, paranoia, panic attacks, increased aggression, or choreoathetoid movements.

TABLE 2
Adverse Effects

	%
Decreased desire to perform mental or physical tasks	70
Decreased appetite	65
Trismus	50[a]
Decreased libido	45
Increased restlessness/agitation	35
Bruxism	30
Increased anxiety	25
Decreased ability to perform mental or physical tasks	20
Disorientation/confusion	15[b]
Nausea/vomiting	15
Increased fear	15
Increased defensiveness	15
Decreased ability to interact with or be open with others	10
Depressed mood	10
Nystagmus	10[c]
Increased obsessiveness	5
Motor tics	5[d]
Increased compulsiveness	5
Headaches	5
Decreased awareness of emotions	5
Increased aggression	0
Auditory hallucination	0
Choreoathetosis	0
Panic attacks	0
Paranoia	0

[a]One subject reported that he experienced chronic trismus prior to taking MDMA. While taking MDMA, the trismus stopped, but it returned again once the drug effect wore off.

[b]Two of the subjects who reported disorientation or confusion emphasized that this happened to them during only one of multiple experiences with MDMA and, in both subjects, extenuating circumstances were present. One subject had combined MDMA with marijuana. The other was in the mountains at the time and had been experiencing disorientation prior to taking MDMA. The MDMA intensified the disorientation.

[c]This symptom was not specifically asked about but was described by two subjects.

[d]Subject described trembling of face and hands and hyperreflexia.

Sequelae

Sequelae were divided into short term (less than 1 week) and long term (greater than 1 week). This division was an arbitrary one made after the data were obtained to assist with interpretation. The short- and long-term sequelae are listed in Tables 3 and 4. The majority of these sequelae are self-explanatory. However, a few deserve further description.

Changes in values or life priorities often involved a shift away from materialistic values and toward interpersonal relationships. Quotations included: "increased priority on spiritual matters and relationships"; "less materialistic . . . more interested in quality of life"; "increased focus on relationships"; "less materially oriented . . . keeping things simple . . . showing compassion for other people"; "further confirmation of spiritual orientation"; "more focused on education and learning"; "heightened prioritizing of aesthetic values"; and "less driven . . . more contented."

Among the six subjects who reported long-term changes in their sense of separation from others, five described more persistent alterations and the sixth described changes that lasted only a few weeks. All six subjects stated that these changes involved an experience of feeling less separate from others. This was not described as problematic or as a psychotic fusion in which the boundaries between self and other were nonexistent, but instead as a positive state in which the boundaries were less rigid and impermeable and an enhanced sense of empathy existed. Thirty percent (6 of 20) of subjects also reported an increased interest in religious issues and commitment to spiritual practices.

Six subjects reported ongoing medical problems at the time they took MDMA. Of these, 33% (2 of 6) reported changes in their medical problems following the use of MDMA. These included decreased pain in a chronic arthritic condition and an increased dedication to physical therapy for treatment of chronic back pain.

All subjects denied any craving (defined as "a need or compulsion") to take MDMA again, but 70% (14 of 20) reported that they "had an interest" in taking it again. Of the 30% (6 of 20) who did not have an interest, half (3 of 6) had previously had unpleasant or dysphoric experiences with MDMA.

Subjects were then asked "Would you use MDMA again?" and 85% (17 of 20) responded that they would. Among the three subjects who stated they would not use MDMA again, two described prior dysphoric or unpleasant experiences; "it didn't feel good" and "I don't like what it does to my body . . . it doesn't transform you" were characteristic quotations. The third

TABLE 3
Short-Term Sequelae

	%
Decreased sleep	40
Decreased appetite	30
Increased sensitivity to emotions	25
Decreased ability to perform mental or physical tasks	20
Decreased desire to perform mental or physical tasks	20
Increased ability to interact with or be open with others	20
Decreased defensiveness	20
Fatigue	15[a]
Decreased aggression	15
Decreased fear	15
Cognitive changes	15
Depressed mood	10
Decreased obsessiveness	10
Speech changes	10
Increased restlessness/agitation	10
Altered perception of time	10
Decreased anxiety	10
Decreased libido	10
Trismus	10[b]
Change in ego boundaries	5
Decreased restlessness/agitation	5
Increased appetite	5
Decreased impulsivity	5
Nausea/vomiting	5
Decreased memory	5
Bruxism	5
Decreased compulsiveness	5
Increased libido	5

[a]This symptom was not specifically asked about, but it was mentioned by three subjects.
[b]Includes one subject with pre-existing trismus.

TABLE 4
Long-Term Sequelae

	%
Improved social/interpersonal functioning	50
Changes in religious/spiritual orientation or practice	46[a]
Changes in values or life priorities	45
Improved occupational functioning	40
Increased ability to interact with or be open with others	35
Decreased defensiveness	30
Changes in ego boundaries	30
Decreased desire to use alcohol	25
Decreased fear	20
Increased sensitivity to emotions	15
Increased desire to use hallucinogenic substances	15
Improved family relationships	15
Change in career plans	15
Decreased restlessness/agitation	10
Decreased obsessiveness	10
Decreased compulsiveness	5
Increased impulsivity	5
Decreased impulsivity	5
Cognitive changes	5[b]
Decreased aggression	5
Speech changes	5[c]

[a]6 of 13 = 46%.
[b]Subject reported a permanent shift to a more "positive" cognitive set.
[c]Subject described a permanent increase in ability to produce speech.

subject stated that the present illegal status of MDMA was the reason for not taking it again.

Repeated MDMA Use

The 14 subjects who had used MDMA on more than one occasion were questioned about any changes in the nature of their MDMA-induced experience with repeated use. None of the subjects reported using an escalating dose of MDMA and no consistent changes were found with recurrent use in the intensity, pleasure, or insight gained from the experience.

Attitudes toward MDMA

When asked about current attitudes toward MDMA, 85% (17 of 20) of the subjects expressed concern about reported neurotoxic effects of the drug. Despite these concerns, 85% (17 of 20) were in support of further clinical research utilizing MDMA. Among the three subjects who were not in support of research, two expressed ambivalence and the third was unequivocally against research. Of the two ambivalent subjects, one was concerned about the abuse potential of MDMA and the other about possible serotonergic neurotoxicity. The subject who was against research felt that the drug had no therapeutic potential at all. These three subjects had each experienced at least one episode with MDMA which they described as unpleasant.

All subjects reported that they had never given MDMA to a patient.

Discussion

The phenomenology of MDMA can be divided into three categories: (a) perceptual changes; (b) changes in affective state; and (c) changes in interpersonal relationships. Perceptual changes most frequently involved sensory intensification and an altered perception of time.

Affective states showed both quantitative and qualitative changes. Fifty percent of subjects reported increased awareness of their emotions while taking MDMA. Qualitative changes were similar to those previously reported by other authors (*e.g.*, heightened sense of closeness, increased feelings of intimacy, etc. [Greer and Tolbert, 1986; Peroutka et al., 1988]).

Ego boundary changes were generally experienced as reduced distinction between self and not-self. However, this loss of self-other distinction seemed different from the fusion of self and object representations found in schizophrenia and borderline personality disorder states, since subjects found the experience positive,

reported increased empathy, and remained clearly aware of the conventional self-other distinctions as adaptive ego constructs. This combination of increased identification and empathy with others together with awareness of the conventional self-other boundaries has been described as one of the characteristics distinguishing peak or transpersonal experiences from pathological fusion (Nelson, 1990; Shapiro, 1990). Apparently, similar experiences of transcending self-other boundaries have been described across centuries in mystics as well as in contemporary peak, meditative, and some psychedelic experiences (Engler, 1981; Goleman, 1988; Maslow, 1971; Shapiro and Walsh, 1984), although there does exist considerable controversy over whether drug-induced unitive experiences can rightfully be regarded as "true" mystical experiences (Grof, 1980; Smith, 1964; Walsh, 1990). It seems plausible that the combination of altered ego boundaries and decreased fear may be related to the reduction in psychological defensiveness reported by 80% of the subjects. When people feel more "connected" with others, and experience more self-acceptance (as has been reported previously), there is less need to be defensive.

The adverse effects most frequently reported are similar to those of structurally related compounds. Amphetamine-like effects were most prevalent (*e.g.*, anorexia, trismus, motor restlessness, bruxism). Hallucinatory or illusory phenomena were reported infrequently. Of the four subjects who reported visual phenomena of these types, two combined MDMA with other psychoactive substances (alcohol or marijuana) and the other two reduced visual input by using a blindfold or darkened room. This low incidence of visual illusions and hallucinations is in marked contrast to psychedelics such as LSD.

It is interesting to note that 70% (14 of 20) of subjects reported a decreased desire to perform mental or physical tasks during the MDMA experience, which was twice as common as subjects reporting motor restlessness. This is in contrast to the increased activity generally observed with amphetamines. Further investigation is needed to clarify the nature of this change.

Another notable finding from this study was that while short-term sequelae included both adverse effects and changes in interpersonal functioning, the most commonly reported long-term sequelae involved improved functioning (*e.g.*, interpersonal, occupational) and changes in attitudes or behaviors (*e.g.*, religious/spiritual orientation or practice, values, life priorities). In fact, the list of reported long-term sequelae was notable for its lack of deleterious effects. These findings stand in marked contrast to many popular and professional assumptions regarding MDMA use. Factors that may have contributed to the unexpectedly low number of adverse effects include: the high functional status of the subjects, the high number of subjects who prepared themselves in some way prior to using MDMA, careful structuring of the environment, and the relatively high frequency of positive expectations.

This study found no changes in the effects of MDMA with repeated use. One subject reported taking the drug on at least 25 different occasions over 4 years with no reduction in the drug's effectiveness and no increase in dosage utilized. This contrasts with previous reports of reduced efficacy with repeated use of MDMA (Peroutka, 1989). It is noteworthy that the subject just mentioned used MDMA at intervals separated by a minimum of several weeks.

Over half of the subjects studied stated that they believed the MDMA experience has a high or very high potential as an adjunct for pychotherapy, particularly in regards to its capacity to enhance empathy. This is consistent with the reports of some investigators who have identified MDMA as an empathogen (Adamson and Metzner, 1988). Concern was also raised, however, over the potential for abuse of MDMA. The uncontrolled use of MDMA, especially by young people, with poor appreciation of set and setting, mixing MDMA with alcohol or other drugs, and disregarding proper safety standards were all cited as concerns over the potential for dangerous misuse of MDMA.

There are several methodological limitations associated with a study of this type. First, the sample was not randomly selected. The reports obtained were subjective and had no objective measurements to confirm them. While objective physiological and behavioral measures are possible, they would necessitate a study of a different nature. The attempt here was to focus on phenomenology, since so little is known about the MDMA user's personal experience.

Another limitation is that the reports are retrospective, which means that they may be distorted by inaccurate recollections and biases of the subjects. An additional problem is that the subjects reported on their use of what is now an illegal substance and, therefore, may have been inclined to delete or alter their reports. Yet subjects were found to be very willing to describe their experiences. Finally, there are many variables that could not be controlled, *e.g.*, dose and purity of MDMA, number of times MDMA was used, set and setting, and the time between interview and the last use of MDMA. Despite these limitations, the data provide much useful information about the effects of this drug.

It appears that MDMA induced an alteration in consciousness that most subjects felt was pleasant and valuable, although a smaller number of subjects reported a temporary dysphoria with no lasting benefits. Although most subjects supported clinical research utilizing MDMA, many also expressed concerns about the

potential for long-term serotonergic neurotoxicity as well as its history of indiscriminate use in the population at large. Clearly, this unique chemical compound raises many intriguing basic science and clinical questions. Although the future legal status of MDMA and, therefore, the possibility of rigorously investigating its clinical properties and potentials remain unknown, we encourage fresh review of existing data as well as further investigation of nontoxic analogues of MDMA.

References

Adamson S, Metzner R (1988) The nature of the MDMA experience and its role in healing, psychotherapy, and spiritual practice. *Revision* 10:59–72.

Barnes DM (1988) New data intensify the agony over ecstasy. *Science* 239:864–866.

Barron SP, Lowinger P, Ebner E (1970) A clinical examination of chronic LSD use in the community. *Compr Psychiatry* 11:69–79.

Battaglia G, Yeh SY, DeSouza EB (1988) MDMA-induced neurotoxicity: Parameters of degeneration and recovery of brain serotonin neurons. *Pharmacol Biochem Behav* 29:269–74.

Blacker KH, Jones RT, Stone GC, Pfefferbaum D (1968) Chronic users of LSD: The "acid heads." *Am J Psychiatry* 125:341–351.

Commins DL, Vosmer G, Virus RM, Woolverton WL, Schuster CR, Seiden LS (1987) Biochemical and histological evidence that methylenedioxymethylamphetamine (MDMA) is toxic to neurons in the rat brain. *J Pharmacol Exp Ther* 241:338–345.

Derome-Tremblay M, Nathan C (1989) Fenfluramine studies. *Science* 243:991.

Dowling GP, McDonough ET, Bost RO (1987) 'Eve' and 'Ecstasy.' A report of five deaths associated with the use of MDEA and MDMA. *JAMA* 257:1615–1617.

Downing J (1986) The psychological and physiological effects of MDMA on normal volunteers. *J Psychoactive Drugs* 18:335–40.

Engler J (1981) Therapeutic aims in psychotherapy and meditation: Developmental stages in the representation of self. In J Engler, K Wilber, D Brown (Eds), *Transformations of consciousness.* Boston: New Science Library.

Goleman D (1988) *The meditative mind.* Los Angeles: Tarcher, Inc.

Greer G, Strassman R (1985) Information on "Ecstasy." *Am J Psychiatry* 142:1391.

Greer G, Tolbert R (1986) Subjective reports of the effects of MDMA in a clinical setting. *J Psychoactive Drugs* 18:319–327.

Grinspoon L, Bakalar J (1979) *Psychedelic drugs reconsidered.* New York: Basic Books.

Grinspoon L, Bakalar J (1986) Can drugs be used to enhance the psychotherapeutic process? *Am J Psychother* 40:393–404.

Grob C, Bravo G, Walsh R (1990) Second thoughts on 3,4-methylenedioxymethamphetamine (MDMA) neurotoxicity. *Arch Gen Psychiatry* 47:288.

Grof S (1980) *LSD psychotherapy.* Pomona, CA: Hunter House.

Lawn J (1988a) Schedules of controlled substances: Deletion of 3,4-methylenedioxymethamphetamine (MDMA) from schedule 1 of the Controlled Substances Act. *Fed Reg* 53:2225.

Lawn J (1988b) Schedules of controlled substances: Scheduling of 3,4-methylenedioxymethamphetmaine (MDMA) into schedule 1 of the Controlled Substances Act; Remand. *Fed Reg* 53:5156.

Logan B, Laverty R, Sanderson W, Yee YB (1988) Differences between rats and mice in MDMA (methylenedioxymethylamphetamine) neurotoxicity. *Eur J Pharmacol* 152:227–234.

Maslow A (1971) *The farther reaches of human nature.* Viking: New York.

McGlothlin W, Cohen S, McGlothlin MS (1967) Lasting effects of LSD on normals. *Arch Gen Psychiatry* 17:521–532.

Nelson JE (1990) *Healing the split: Madness of transcendence.* Los Angeles: JP Tarcher.

Peroutka S (1987) Incidence of recreational use of 3,4-methylenedimethoxymethamphetamine (MDMA, "Ecstasy") on an undergraduate campus. *N Engl J Med* 317:1542–1543.

Peroutka SJ (1989) Ecstasy: A human neurotoxin? *Arch Gen Psychiatry* 46:191.

Peroutka SJ, Newman H, Harris H (1988) Subjective effects of 3,4-methylenedioxymethamphetamine in recreational users. *Neuropsychopharmacology* 1:273–277.

Pohlman AD (1957) The use of D-amphetamine sulfate as an adjunct to psychotherapy. *J Clin Exp Psychopathol* 18:156–159.

Price LH, Ricaurte GA, Krystal JH, Heninger GR (1989) Neuroendocrine and mood responses to intravenous L-tryptophan in 3,4-methylenedioxymethamphetamine (MDMA) users. *Arch Gen Psychiatry* 46:20–22.

Ricaurte G, Bryan G, Strauss L, Seiden L, Schuster C (1985) Hallucinogenic amphetamine selectively destroys brain serotonin nerve terminals. *Science* 229:986–988.

Ricaurte GA, Forno LS, Wilson MA, DeLanney LE, Irwin I, Molliver ME, Langston JW (1988) 3,4-Methylenedioxymethamphetamine selectively damages central serotonergic neurons in non human primates. *JAMA* 260:51–55.

Savage C, Fadiman J, Mogar R, Allen MH (1966) The effects of psychedelic (LSD) therapy on values, personality, and behavior. *Int J Neuropsychiatry* 2:241–254.

Savage C, Savage E, Fadiman J, Harman W (1964) LSD: Therapeutic effects of the psychedelic experience. *Psychol Rep* 14:111–120.

Schmidt CJ (1987) Neurotoxicity of the psychedelic amphetamine, methylenedioxymethamphetamine. *J Pharmacol Exp Ther* 240:1–7.

Schmidt CJ, Taylor VL (1987) Depression of rat brain tryptophan hydroxylase activity following the acute administration of methylenedioxymethamphetamine. *Biochem Pharmacol* 36:4095–4012.

Schuster CR, Lewis M, Seiden LM (1986) Fenfluramine neurotoxicity. *Psychopharmacol Bull* 22:148–151.

Seymour RB (1986) *MDMA.* San Francisco: Haight-Ashbury Publications.

Shapiro D (1990) A sense of control, health, and illness: Exploring the mind body relationship and the social-cultural/spiritual context. *Int J Psychosomatics* 37:40–49.

Shapiro D, Walsh R (Eds) (1984) *Meditation: Classic and contemporary perspectives.* New York: Aldine.

Sherwood JN, Stolaroff MJ, Harman WW (1962) The psychedelic experience—A new concept in psychotherapy. *J Neuropsychiatry* 4:69–81.

Smart RG, Jones D (1970) Illicit LSD users: Their personality characteristics and psychopathology. *J Abnorm Psychol* 75:286–292.

Smith H (1964) Do drugs have religious import? *J Philosophy* 61:517–530.

Stone DM, Hanson GR, Gibb JW (1987) Differences in the central serotonergic effects of methylenedioxymethamphetamine (MDMA) in mice and rats. *Neuropharmacology* 26:1657–1661.

Unger SM (1963) Mescaline, LSD, psilocybin, and personality change. *Psychiatry* 26:111–125.

Walsh R (1990) *The spirit of shamanism.* Los Angeles: JP Tarcher.

Walsh R, Vaughan F (Eds) (1980) *Beyond ego.* Los Angeles: JP Tarcher.

Welpton DF (1968) Psychodynamics of chronic lysergic acid diethylamide use. *J Nerv Ment Dis* 147:377–385.

Whitaker-Azmitia PM, Aronson TA (1989) "Ecstasy" (MDMA)-induced panic. *Am J Psychiatry* 146:119.
