# Supplementary material for: Protocol for a qualitative mechanistic study of MDMA with a sample of psychoanalytic psychotherapists: A phenomenological investigation
Source: PLoS One. 2026 Jun 18;21(6):e0350728. doi: 10.1371/journal.pone.0350728 (PMC13278426; doi:10.1371/journal.pone.0350728)
Supplement: Attachment — Submitted filename: lehrnerYehuda2026structured-protocols-in-mdma.pdf [file pone.0350728.s003.pdf]

Check for updates

Psychedelic Medicine
Volume 00, Number 00, 2026
Mary Ann Liebert, A Part of Sage
© The Author(s)
DOI: 10.1177/28314425261429233

psychedelic medicine

COMMENTARY

Balancing Innovation and Evidence: Reflections on Structured Protocols in MDMA-Assisted Therapy Versus a Principle-Guided, Patient-Directed Approach

Amy Lehrner,[1,2,*] Miryam Sperka,[1,2] Lauren A. Lepow,[1] Laura C. Pratchett,[1,2] Tamar Glatman Zaretsky,[1] Mustafa Mehmet Haznedar,[1,2] and Rachel Yehuda[1,2]

Abstract
There is growing interest in incorporating MDMA (3,4-Methylenedioxymethamphetamine) into structured, evidence-based cognitive-behavioral treatment protocols for post-traumatic stress disorder. Yet this risks overlooking the distinctive qualities of MDMA-assisted therapy, which has already demonstrated strong clinical effects, high response rates, and low dropout using a principle-guided, patient-directed therapeutic model. This model integrates MDMA administration into a broader therapeutic process, including nondrug preparatory and integrative sessions, designed to support the unique, nonlinear experience often elicited by MDMA. This perspective paper outlines the theoretical and clinical rationale for preserving and refining the patient-directed model, arguing that it represents a distinct innovation in trauma treatment that is not well suited to existing protocols. Attempting to embed MDMA within highly structured, manualized treatment protocols may misapprehend the synergistic nature of the therapy, blunt its most transformative effects, and even potentially cause harm. Rather than immediately turn to existing models, the field should prioritize research investigating real-world evidence, treatment optimization, and mechanisms of action of this new model.

Keywords: MDMA, psychedelic, MDMA-assisted therapy, post-traumatic stress disorder, psychotherapy

There is a growing interest in integrating MDMA into established, highly structured trauma-focused psychotherapies for posttraumatic stress disorder (PTSD), such as Prolonged Exposure and Cognitive Processing Therapy.[1,2] The rationale behind such combinations is that these protocols have already demonstrated efficacy, and the addition of MDMA might enhance treatment tolerability and improve outcome. Some argue that combining a new medication—especially a controversial one like MDMA—with an existing structured and manualized trauma therapy is a more sound clinical and scientific approach.[3] These therapies represent the dominant paradigm in PTSD treatment and are the primary focus of academic research. It makes sense prima facie to propose pairing a novel drug for PTSD with the gold-standard psychotherapies that have proven effectiveness.

The argument for combining these psychotherapies with MDMA, however, may partly stem from unfamiliarity with the radically different effects and needs engendered by the use of MDMA. As a team of clinicians with decades of experience providing and training students in the use of manualized treatments for PTSD, including PE[4] and CPT,[5] we have a deep appreciation for these therapies, the valuable impact they can have on patients, and the theoretical foundations of the treatments. We are in the relatively rare position of also having had the opportunity to provide MDMA-assisted therapy to the same population of patients with PTSD in the context of clinical trials for the past

[1]The Parsons Center for Psychedelic Healing, Icahn School of Medicine at Mount Sinai, New York, New York, USA.
[2]James J. Peters VA Medical Center, New York, New York, USA.

*Address correspondence to: Amy Lehrner, PhD, The Parsons Center for Psychedelic Healing, Icahn School of Medicine at Mount Sinai, 1 Gustave Levy Place, New York, NY 10029, USA, E-mail: Rachel.yehuda@mssm.edu

5 years. It is from our experiences with these different modalities that we share our speculations and concerns about implementing an agenda-driven therapy in conjunction with MDMA. This is a critical time in the field as different approaches are being studied and discussed, and we propose that MDMA represents a distinct innovation in trauma treatment that is not well suited to concurrent use with highly structured and agenda-driven protocols.

A Different Therapeutic Paradigm

Consistent with most trauma-focused therapies, MDMA-assisted therapy theorizes that PTSD reflects a disruption of a natural recovery process and that treatment must create healing conditions in which individuals can engage with, rather than avoid, distressing trauma-related material. The central difference from manualized CBT treatments, however, is in how the optimal healing conditions are conceptualized and actualized in the context of MDMA administration. Rather than approaching MDMA dosing sessions and subsequent therapy as distinct, independent components of an additive treatment, it is our belief that the therapy and the medication together represent an integrative, synergistic treatment that is greater than the sum of its parts.[6–8] The synergy emerges from the integration of the catalytic experience of MDMA into a therapeutic container that privileges the emergence and exploration of clinical material *as it arises from the patient*. This patient-directed approach (also called an inner-directed model) has been the central principle of MDMA-assisted therapy and represents a significant difference from structured trauma-focused therapies.

Manualized Trauma Therapies: Theoretical Underpinnings and Structural Constraints

This approach shares some important theoretical and methodological elements with evidence-based structured treatment protocols while potentially minimizing some of the challenges that can make these treatments difficult to tolerate and may contribute to their limited effectiveness for many patients (e.g., focused on a single index trauma).[9–11] Highly structured treatments like PE and CPT follow detailed, time-limited protocols that place the therapist in a directive role. While the process of identifying the trauma content of focus can be quite collaborative, determining the topic and focus of each session, as well as the specific skills introduced, follows a more set and directed structure.

For example, in PE, the patient is directed to engage in imaginal exposure to a single traumatic event, repeatedly narrating the experience from start to finish for 30–60 min. The narration is recorded and assigned as daily homework between sessions. In CPT, the patient also selects a single traumatic event and evaluates beliefs about why the event happened, what it means about themselves, others, and the world, and how it has affected them. The therapist helps the patient identify "stuck points"—maladaptive beliefs or cognitive distortions theorized to maintain their PTSD (e.g., "if I had fought back I could have prevented the assault," or "I am a monster for what I did during combat"). Once a list of stuck points is generated, the patient learns how to evaluate and challenge these thoughts using a series of structured worksheets. CPT then progresses through a series of PTSD-related topics—safety, trust, power/control, self-esteem, and intimacy—each introduced with handouts and worksheets in a session-by-session sequence. There is space in each of these approaches for collaboration and individualization; the particular memory for imaginal exposure, the hierarchy for in vivo exposures, identification of stuck points, and the material that arises from these interventions are all unique to each patient. However, this occurs within a highly structured framework and with direction that deviation from the set structure is not evidence-based and can decrease efficacy. While there are adapted versions of the CPT handouts, even with these, the overall steps for addressing maladaptive cognitions remain the same.

PE is based on emotional processing theory, which posits that PTSD is maintained by avoidance of trauma-related thoughts, feelings, and situations. This avoidance reinforces pathological fear structures and prevents fear from being processed and resolved. The primary intervention in PE is exposure, which supports habituation and inhibitory extinction learning, and processing of trauma-related emotions and cognitions. In this way, PE emphasizes "bottom-up" processes activated by behavioral changes—specifically, direct exposure to feared stimuli.

CPT, while also informed by emotional processing theory, draws on social cognitive theory, which emphasizes the universality of people's efforts to make meaning after trauma. CPT thus emphasizes "top-down" cognitive strategies, with change theorized to occur through cognitive restructuring—helping patients challenge and modify extreme or distorted beliefs in favor of more accurate and balanced interpretations of the traumatic event.

Manualized treatments that implement a structured series of interventions in a predetermined manner and order are easier to replicate and study, since therapists follow the same session agendas. However, they are therapist-directed and highly structured, often allow little time for trust-building, and typically require identification of a single trauma and structured engagement with that material. These models include collaboration to the extent that patients provide the content for exposure and cognitive restructuring, but the treatments themselves are fundamentally guided by a preset agenda with specified techniques. In practice, many therapists adapt these protocols by adjusting pacing, addressing comorbid conditions or psychosocial stressors, or responding to cultural and relational dynamics. That these adaptations are often

needed underscores the constraints of rigid manuals and the need for clinicians to respond flexibly to patient needs.

In many ways, the theorized mechanisms of change in MDMA-assisted therapy are consistent with these approaches. Like PE, CPT, and other evidence-based psychotherapies (EBPs), MDMA-assisted therapy emphasizes emotional engagement, trauma processing, and exploration of how patients make meaning of their experiences. Therapists practicing MDMA-assisted therapy must understand the theoretical underpinnings of trauma-focused therapies, as prevailing theories of PTSD development and maintenance inform therapeutic interventions throughout the process. A crucial difference, however, lies not only in what happens during therapy but how it unfolds.

The Healing Process in MDMA-Assisted Therapy

In essence, we have found that while therapists may draw on techniques of PE, CPT, or other EBPs, the effects of MDMA often appear to open a healing process in which patients intuitively know what they need. MDMA seems to catalyze recovery much like immunotherapy in medicine—by activating the body's own internal healing mechanisms. While common mechanisms such as emotional approach, tolerance, and curiosity appear central to change, MDMA opens multiple pathways to recovery. When therapists do not privilege any single mode—cognitive, behavioral, affective, or somatic pathways—patients are free to follow whatever MDMA evokes. We have had patients with vivid metaphorical visual experiences, for example, of providing comfort to younger versions of themselves, of connection with the cosmos and nature, and of taking a train away from a version of self trapped in self-hatred. These powerful images have emerged in the context of a patient-directed MDMA model, where patients were encouraged to be open to whatever was coming up from inside of themselves. A manualized, directed model risks constraining such opportunities or requiring patients to turn to other material instead of these experiences and thus may bypass the most powerful potential for healing offered by MDMA.

In some cases, patients revisit traumatic memories in ways that are similar to imaginal exposures in PE. Others begin to question and reframe long-held beliefs that have maintained their PTSD. During integration sessions, some have even used metaphors similar to those used in CPT, such as organizing mental "files." But not all patients need to engage in these processes to recover, and their paths are rarely predictable or linear. We have also seen patients improve significantly with minimal trauma narration and others whose healing was primarily somatic and emotional, rather than cognitive.

A significant difference from other forms of therapy is that during MDMA sessions, much of the process happens internally within the patient rather than through therapeutic dialogue that guides or interprets the patient's experience. Patients may engage with therapists for help if they are stuck, to share an insight, or for support during intense emotional processing, but often the process is nonverbal and interior. This process, catalyzed by MDMA, unfolds in the context of a safe therapeutic container that provides support, trust in the process, attunement to emotional needs and engagement, and empathic presence but occurs without direction from the therapist.

The Effects of MDMA and a Patient-Directed Model

In determining a therapeutic approach to use with MDMA, it is essential to account for the unique characteristics of the experience occasioned by the drug's subjective effects. The shifts in consciousness, perception, and emotion that MDMA produces prime a person for unfettered emotional exploration. This process is nonlinear, varies significantly from person to person and from session to session, and the emergent clinical material cannot be predicted. While trauma-informed care assumes that engaging with trauma-related emotions is central to healing, the timing and form of that processing across the course of treatment varies greatly across patients. In MDMA sessions, reconnecting with feelings of positivity, joy, hope, or self-compassion may be just as vital. In our experience, maintaining a therapeutic stance that honors the material emerging at any given moment—trusting that it arises for a reason—has been highly effective in supporting recovery. When therapists remain responsive and help patients trust their own innate capacity to heal, the therapeutic process becomes one that emerges organically from within the patient.

Patients are often surprised by the material that comes up during MDMA sessions, only later to realize its importance. Many find themselves processing childhood memories that shaped their sense of themselves and others and their coping strategies. For example, patients have come to understand how experiences of childhood abuse or witnessing violence against a parent taught them to suppress their emotions, mistrust those who are supposed to provide safety, and adopt roles of protectors or caregivers. Such insights have been pivotal in enabling them to process emotions and face difficult memories. For some, these childhood memories have preceded adult trauma in the therapeutic process; for others, they emerged later. Still others have had a purely somatic experience of processing anxiety, a form of self-directed interoceptive exposure, before any memories or cognitions surfaced.

The Heightened Need for Relational Safety

Introductory sessions with manualized therapies provide psychoeducation about PTSD, explanation of the treatment rationale, collection of trauma history, and early

building of trust. These elements align well with MDMA preparation. However, MDMA work also demands deeper attention to emotional and relational safety. Taking MDMA can feel like stepping off a cliff, not knowing what will happen or how one will land. This can be terrifying and a serious trust fall, especially for individuals who have been traumatized. Preparation that relies too heavily on didactic teaching risks reinforcing the expert-patient hierarchy and may fall short in creating the foundations of safety needed to fully engage with the medicine. If patients do not feel safe with their therapists, we would argue that they should not proceed to an MDMA session.

In addition to the opportunities MDMA provides for unpredictable and idiosyncratic clinical material to emerge, MDMA can evoke profoundly intense, emotional, and relational experiences that require heightened attention to physical, emotional, and relational safety and therapist presence and genuineness. Patients may experience increased feelings of safety, trust, and connection, as well as highly vulnerable emotions and memories. They are reclining, listening to music, and engaged in periods of introspection with eyes closed or wearing eyeshades. In our experience, patients in the altered state induced by MDMA require a level of attunement, presence, genuineness, and support that is different from other forms of therapy. While research has demonstrated the relevance of the therapeutic alliance across modalities, MDMA can place the therapeutic relationship as a central nexus of healing, rather than merely a tool for engagement. This can be especially true for patients with developmental or attachment wounds.

MDMA can enhance the patient's sensitivity to relational cues, enhance transferential dynamics, and lower protective defenses that keep patients from sharing deeply sensitive experiences and asking for support from their therapist. For example, a patient revisiting a series of neglectful and abusive experiences during childhood that included feelings of shame and unworthiness reached out for the therapist's hand and said that they needed to take in some "mother energy." The empathic presence of a therapist, responding affirmatively to an expression of need from a patient who had been shamed for this by their own mother and holding the patient's hand while they processed their feelings, was an essential part of their recovery of a sense of self-worth and value. The patient came to realize during the unfolding therapy process that this feeling of having no value or worth was the underlying theme of their life. The patient-directed model, which allowed the patient to follow these memories and feel sensations, provided the opportunity to realize this pattern, feel it, and have a different and healing experience of self and other that ultimately proved foundational for their recovery trajectory. Had the patient been redirected to focus on only one memory or cognitively work through a single belief about the experience, they would have missed the opportunity for the more embodied, self-directed insight and connection that MDMA allowed; worse, it could have reinforced the patient's beliefs about themselves as unworthy or lesser than those of others.

Patients in our trials have more than once looked at their therapists as they explore feelings of brokenness or self-judgment while in non-ordinary states of consciousness and directly asked why the therapists care about them or what they see in them of worth. There is a longing and a need for empathy, connection, and compassion that is palpable. These moments require therapists to be present not just as professionals and to go beyond the regular training and experience of nonpsychedelic therapy. We have also observed moments when a need for connection went unheeded and the damage and derailment that can occur when a patient feels abandoned or alone in a non-ordinary state.

Challenges of Using MDMA to "Assist" Structured CBT Protocols

There is concern that introducing MDMA into a therapeutic model that directs the patient and dictates the topic and structure of each session could constrain and limit the emergent material and experiences that MDMA generates. This could stifle the breakthroughs that emerge when patients can explore and process trauma-related memories and emotions as they arise organically. Such an approach could result in the suppression, avoidance, or overlooking of important material, emotions, or relational experiences due to the therapist's agenda redirecting patients away from the most clinically relevant material for the patient at that time. For example, we have seen many patients with severe PTSD who had a primary concern that they were unable to engage in relationships, feeling disconnected, broken, and incapable of love. Trauma theory suggests that the patient's disconnection was a reflection of a failure to process the emotions and meanings related to their trauma and subsequent suppression of all emotional experience. As a result, CBT models would encourage the patient to directly engage with the memories and emotions of the trauma in the belief that positive feelings will become accessible after the avoidant (but protective) coping strategies are removed. However, for some of these patients, the first MDMA session has been characterized by feelings of self-compassion, connection, and empathy. Discovering the capacity for these feelings within themselves preceded, rather than emerged from, processing trauma directly. A therapy that had instructed them to focus on trauma first would have missed the opportunity for these transformative experiences, which proved foundational for the patients' ability to face painful memories later in the process.

Practitioners familiar with trauma-focused manualized therapies may worry that the emphasis on following the

patient may risk collusion with avoidance or incomplete processing of trauma memories. There are a few relevant considerations. (1) Because defenses are relaxed under MDMA, it is particularly important that therapists respect the patient's judgment about what they are prepared to engage with and never push beyond the patient's limits (i.e., respecting and deferring to protective defenses). (2) As a result, unlike approaches that treat avoidance as uniformly counterproductive, MDMA-assisted therapy invites consideration of moments of avoidance as a potential form of self-regulation—patients titrating their engagement to match current needs and emotional capacity. The therapists model curiosity about potential avoidance nonjudgmentally, encouraging patients to notice and be curious about their internal experiences and reinforcing the principle that healing unfolds at the patient's pace. (3) One unique aspect of MDMA is the nonlinearity of the experience and therapeutic process. MDMA induces a nonordinary state of consciousness that includes changes in perception, cognition, and mood, which often includes rapid shifts through different memories, emotional states, and thought processes.

To therapists accustomed to linear, stepwise models, this trajectory can appear disorganized, avoidant, or incomplete. However, such variability is expected with MDMA and can be valuable. For example, if a patient suddenly moves from processing one thing to an entirely different experience, the therapist may wonder if this represents avoidance or leaves material only partly processed. While the therapist may hold curiosity about this, in our experience, when the therapist waits and does not intervene prematurely, it often turns out that the shift was simply a nonlinear progression of the person's healing process that benefited from support rather than interruption. Notably, if the patient appears not to be on a recovery trajectory or feels stuck, the therapists may then become more active in scaffolding more sustained emotional engagement.

There are additional challenges of implementing a highly structured therapy in the context of MDMA sessions. For example, PE requires cognitive engagement and deliberate task execution, as well as a focus on a single trauma narrative. These expectations may feel artificial or incongruent when placed within the expansive, non-ordinary nature of the MDMA experience. Participants may feel pressured to remain on task and focused on the identified index trauma, potentially suppressing the very emotional content that MDMA can unlock or leading to a rupture in the therapeutic alliance. Such experiences can be especially problematic for individuals with complex or relational trauma histories, for whom coercion and invalidation are not just past experiences but organizing principles in their lives. Others may struggle to retrieve memories on demand, potentially leading to frustration, shame, or self-blame. In our clinical trials of MDMA-assisted therapy for PTSD, participants often reported that during an MDMA session, they had tried to direct their thoughts or focus on a memory, only to find they could not and that attempts to do so only proved frustrating or derailed their internally driven process. Furthermore, given that most patients treated for PTSD have experienced multiple traumas—including combat veterans and survivors of chronic abuse—focusing on a single "index trauma" is not only limiting but also potentially invalidating. MDMA-assisted therapy offers the chance to process the totality of the impact of trauma and pain, and a therapeutic approach that narrows one's experience to a singular event represents a potential missed opportunity to make use of the unique properties of MDMA for more holistic healing.

The Role of Integration

After MDMA, patients sometimes feel overwhelmed by the experience, often using metaphors of feeling like they were hit with water from a firehose or are in a shaken snow globe. Post-MDMA nondrug therapy sessions (often called "integration") allow the patient to sort through their thoughts and feelings, organizing the experience and making sense of what can feel like rapid and profound shifts in self-concept, emotional experience, and perspectives on traumatic experiences. That the patient's MDMA experience is usually substantially internal highlights the necessity for a patient-directed integration process in which patients can share and reflect on what happened for them, process related emotions, and begin to integrate new insights and meanings into their self-concept and ways of being in the world.[12] These sessions are not unlike the unstructured processing component of PE sessions in which patients reflect on the experience of engaging in imaginal exposure with support but limited direction from the therapist. After MDMA, requiring patients to engage in a predetermined set of interventions (e.g., imaginal exposure to a trauma, psychoeducation, or worksheets on a different topic each session) risks leaving them with potentially destabilizing experiences from MDMA sessions without an opportunity to process them. For example, some patients experience intense revisiting of traumatic experiences during MDMA, after which more open-ended processing is needed, whereas a session asking them to engage with a worksheet on stuck points would be seriously misattuned. In our experience, the process of patient-directed exploration of the meaning and experiences they had during MDMA sessions is often when patients experience significant shifts.

Importantly, many of the presumed mechanisms of change associated with PE and CPT—such as revisiting trauma narratives, approaching and staying with painful emotions, re-evaluating trauma-related cognitions, and changing longstanding habits and behaviors—can occur

spontaneously in MDMA-assisted therapy.[13] But these changes and insights can be driven by the patient's experience and process and, conversely may actually be constrained by implementation of an agenda-driven treatment manual. This distinction has been a crucial difference between MDMA-assisted therapy and our experience of implementing other manualized therapies.

Patient-Directed Does Not Mean Nondirected

The principle of a patient-directed process does not mean that therapists never provide direction and structure, but that therapists first follow the patient's impulse and only intervene after first providing space for the process to resolve itself. Often, once that process is allowed to play out, there is no longer a need for direction. Some patients may need or benefit from additional scaffolding. If a patient appears lost or requests additional structure, this is consistent with a patient-driven approach, as it trusts the patient's instincts about what they need for healing. Therapists are guided by their knowledge of PTSD and the patient's history in making clinical decisions about providing direction, which is always offered as a suggestion or an idea rather than as instruction (with the exception of direction necessary to maintain physical safety).

For example, after a few MDMA sessions in which a patient felt lost and disconnected at times, they requested that the therapists more actively engage by asking questions and encouraging exploration. This proved productive and strengthened the patient's sense that they could know what they needed, ask for it, and then get what they needed. At other times, patients have stayed mostly internally focused throughout the session, letting therapists know they were having a meaningful experience and doing fine. A model that asks therapists to trust this process and remain available for processing the experience when the patient is ready allows for flexibility that can facilitate the therapeutic potential of MDMA.

A flexible approach that allows for evidence-based strategies for treating PTSD is not inconsistent with a patient-directed model. This approach aligns with the emergence in recent years of principle-driven treatments in which the therapist is guided by particular theoretical concepts and uses clinical judgment to flexibly apply a range of interventions to the individual case.[14–16] Third-generation CBT approaches such as mindfulness-based therapies, motivational interviewing, ACT, and existential- and compassion-focused therapies share many of the humanistic principles and approaches that have been found to be effective in MDMA-assisted therapy for PTSD.[17,18] In our experience, such strategies are best used in response to the patient's needs and the emergent clinical material. Once the patient's focus has shifted, therapists can adjust course accordingly, at times pivoting to a strategy from another trauma-focused treatment model.

A patient-directed approach empowers traumatized individuals in the process of healing, which is key to establishing safety, particularly in the context of the nonordinary state where patients may feel more vulnerable, and establishes a base from which healing will continue after their course of therapy concludes. Allowing the patient to determine their own boundaries, what they want to address and when helps them to maintain a window of tolerance for revisiting trauma-related material on their terms in order to accomplish the emotional processing that is universally understood as central to healing from trauma. It can also provide a reparative experience in contrast to potential past experiences in which boundaries or autonomy were violated or dismissed and create a new experience in which these are honored and validated. Many patients in our trials have completed other trauma-focused therapies and often comment unbidden on the sense of agency and respect that they felt from the patient-directed approach. It is also worth noting that we have treated many patients with MDMA-assisted therapy who would never have engaged in PE or CPT because they could not tolerate direct engagement with traumatic memories or emotions. On the other hand, after MDMA-assisted therapy, some patients have decided to engage in treatments such as marital therapy, mindfulness groups, and values-based therapies such as ACT, with a new perspective and sense of their needs that allows them to engage in ways that were previously untenable for them.

Trials attempting to incorporate MDMA into CBT protocols may revise those protocols to allow for a more patient-directed process during dosing and postdosing therapy sessions; this additional flexibility, while in essence creating a new protocol to test, may help address some of the concerns raised regarding incorporating MDMA into agenda-guided protocols. Whether the addition of prescribed topics and interventions improves outcomes and is acceptable to patients, or instead attenuates efficacy, remains to be determined. If these variations ultimately allow for an unstructured MDMA session followed by agenda-driven therapy sessions, without adequate attention to processing the experiences and insights from the session, such a protocol in effect creates two separate therapeutic experiences and risks missing the healing opportunities opened by the MDMA experience.

The Need for Innovation: A Patient-Directed, Principle-Guided Approach

There have been no significant advances in psychotherapies or pharmacotherapies developed for PTSD since the late 1980s, despite the high social, economic, and public health burden of PTSD. In this context, we argue that there is a desperate need for innovation, such as that presented by a patient-directed MDMA treatment paradigm.[19] In the debate about what approaches are considered "evidence-

based," it is relevant to note that in MDMA-assisted therapy for PTSD clinical trials, the patient-directed therapy alone in the placebo arms, even in the context of disappointment at not receiving MDMA, resulted in reductions in PTSD symptoms that are comparable to or exceed those of the gold-standard treatments for PTSD and had significantly lower dropout rates. MDMA-assisted therapy not only includes 12 nondrug therapy sessions, similar to PE and CPT, but also includes 3 daylong dosing sessions, and therefore more hours of treatment. While therapy dose may contribute to these outcomes (although the literature on this is mixed[20,21] and increasing the doses of PE does not necessarily improve outcomes[22]), the point here is that a patient-directed, nonagenda-driven approach has shown comparable efficacy, and potentially higher tolerability, than other best practice models. Lower dropout rates in MDMA-assisted therapy trials thus far may be due to patients feeling more agency and empowerment in the model or other factors affecting tolerability and acceptability; these factors remain to be investigated.

Our current EBPs for PTSD have been game-changing additions to the treatment toolbox. Many patients have been able to reduce their suffering and increase functioning as a result of these treatments, and some even experience full recovery. We have seen these changes firsthand. But we also acknowledge that for many, these treatments are too difficult to tolerate or leave patients still struggling with significant symptoms. The impulse to pair a structured trauma-focused therapy with MDMA reflects an assumption that MDMA will enhance the efficacy of these existing treatment models. However, there has been less discussion of how these therapies would optimize the therapeutic effects of MDMA. Based on our experience, the pairing seems like a less-than-ideal match. Introducing MDMA into previously established treatment protocols creates new treatments that require significant adaptations —models that are not yet evidence-based for treating PTSD with MDMA. The assumption that these pairings are safe and somehow better is not based on data. EBPs for PTSD may be our strongest existing treatments, but it does not follow that there are no possibilities for powerful new treatments.

We worry that the push to reject the patient-directed model and only promote research on using MDMA to enhance existing, more therapist-directed and structured models risks missing an important opportunity for innovation and an expansion of our current menu of treatment options. Patients need more options, not fewer. New treatments can only become evidence-based if they are tested so that evidence can be amassed and evaluated. There is much to learn about MDMA-assisted therapy and a need for research on real-world evidence, treatment optimization, and mechanisms of action. Video, audio, and transcript data from previous trials offer rich material for qualitative and quantitative mechanistic and process-based research. There are questions about predictors of outcome, treatment course, and key active ingredients.

As we move forward, the new therapeutic possibilities extended by MDMA challenge us to remain open—to the process, to the patient, and to the possibility that something genuinely new is possible. That will require rigorous science and also epistemic humility, curiosity, and a willingness to experiment with new treatment models and research methods. By fostering a holistic, patient-centered treatment process that attends to the psychological, emotional, cultural, relational, and spiritual dimensions of each patient, MDMA-assisted therapy offers healing that has the potential to go beyond symptom resolution to true post-traumatic growth. The field is at an inflection point and faces an opportunity—and responsibility—to understand and develop treatments that are truly designed to maximize the unique potential of this medicine. This is not only about improving outcomes but also about exploring the possibility of a fundamentally different therapeutic process.

Author Disclosure Statement

No competing financial interests exist.

Funding Information

Funding from the Bob and Renee Parsons Foundation and The Steven and Alexandra Cohen Foundation supported the clinical trials that informed this work.

References

1. Rothbaum BO, Schnurr PP. A new generation of psychedelic-assisted psychotherapy: The example of combining MDMA with trauma-focused cognitive-behavioral therapy for posttraumatic stress disorder. Cogn Behav Pract 2025;32(3):393–396.
2. Yaden DB, Earp D, Graziosi M, et al. Psychedelics and psychotherapy: Cognitive-behavioral approaches as default. Front Psychol 2022;13:873279.
3. Weintraub MJ, Miklowitz DJ. How should psychotherapy proceed when adjoined with psychedelics? World Psychiatry 2024;23(1):157–158.
4. Foa EB, Hembree EA, Rothbaum BO, et al. Prolonged Exposure Therapy for PTSD: Emotional Processing of Traumatic Experiences: Therapist Guide, 2nd ed. Oxford University Press: New York, NY; 2019.
5. Resick PA, Monson CM, Chard KM. Cognitive Processing Therapy for PTSD: A Comprehensive Therapist Manual, 2nd ed. The Guilford Press: New York, NY; 2024.
6. Yehuda R, Lehrner A. Psychedelic therapy—a new paradigm of care for mental health. JAMA 2023;330(9):813–814.
7. Zamaria JA, Fernandes-Osterhold G, Shedler J, et al. Psychedelics assisting therapy, or therapy assisting psychedelics? The importance of psychotherapy in psychedelic-assisted therapy. Front Psychol 2025;16:1505894.
8. Sottile RJ, Vida T. A proposed mechanism for the MDMA-mediated extinction of traumatic memories in PTSD patients treated with MDMA-assisted therapy. Front Psychiatry 2022;13:991753.
9. Najavits LM. The problem of dropout from "gold standard" PTSD therapies. F1000Prime Rep 2015;7:43.
10. Zayfert C, DeViva JC, Becker CB, et al. Exposure utilization and completion of cognitive behavioral therapy for PTSD in a "real world" clinical practice. J Traum Stress 2005;18(6):637–645.
11. Hundt NE, Ecker AH, Thompson K, et al. "It didn't fit for me:" A qualitative examination of dropout from prolonged exposure and cognitive processing therapy in veterans. Psychol Serv 2020;17(4):414–421.
12. van der Kolk BA, Wang JB, Yehuda R, et al. Effects of MDMA-assisted therapy for PTSD on self-experience. PLoS One 2024;19(1):e0295926.

13. Feduccia AA, Mithoefer MC. MDMA-assisted psychotherapy for PTSD: Are memory reconsolidation and fear extinction underlying mechanisms? Prog Neuropsychopharmacol Biol Psychiatry 2018;84(Pt A):221–228.
14. Eubanks CF, Goldfried MR. A principle-based approach to psychotherapy integration. In: Handbook of Psychotherapy Integration. Oxford University Press; 2019; pp. 88–104.
15. Levitt HM, Neimeyer RA, Williams DC. Rules versus principles in psychotherapy: Implications of the quest for universal guidelines in the movement for empirically supported treatments. J Contemp Psychother 2005;35(1):117–129.
16. Babl A, Eubanks CF, Goldfried MR. A 21st century principle-based training approach to psychotherapy: A contribution to the momentum of transtheoretical work. Clin Psychol Eur 2024;6(Spec Issue):e11925.
17. Hayes SC, Hofmann SG. "Third-wave" cognitive and behavioral therapies and the emergence of a process-based approach to intervention in psychiatry. World Psychiatry 2021;20(3):363–375.
18. Schneider KJ, Längle A. Introduction: The renewal of humanism in psychotherapy—A roundtable discussion. In: The Handbook of Humanistic Psychology: Theory, Research, and Practice, 2nd ed. Sage Publications, Inc: Thousand Oaks, CA; 2015; pp. 368–372.
19. Yehuda R, Lehrner A, Sperka M, et al. The case for an integrative model: Hypotheses and rationale for Integrative MDMA-Assisted Psychotherapy (IMAP). Psychedelics 2026;3:100008.
20. Juul S, Jakobsen JC, Jørgensen CK, et al. The difference between shorter-versus longer-term psychotherapy for adult mental health disorders: A systematic review with meta-analysis. BMC Psychiatry 2023; 23(1):438.
21. Ciharova M, Karyotaki E, Miguel C, et al. Amount and frequency of psychotherapy as predictors of treatment outcome for adult depression: A meta-regression analysis. J Affect Disord 2024;359:92–99.
22. Ready DJ, Lamp K, Rauch SA, et al. Extending prolonged exposure for veterans with posttraumatic stress disorder: When is enough really enough? Psychol Serv 2020;17(2):199–206.
