# Supplementary material for: Protocol for a qualitative mechanistic study of MDMA with a sample of psychoanalytic psychotherapists: A phenomenological investigation
Source: PLoS One. 2026 Jun 18;21(6):e0350728. doi: 10.1371/journal.pone.0350728 (PMC13278426; doi:10.1371/journal.pone.0350728)
Supplement: Attachment — Submitted filename: My Comments on MDMA in psychoanalytic psychotherapists.docx [file pone.0350728.s004.docx]

<Perhaps the most crucial one was that the trials’ design (MDMA + psychotherapy vs placebo + psychotherapy) did not disentangle drug from psychotherapy effects and did not establish whether psychotherapy is necessary for clinical benefits, thus creating an interpretability problem. Moreover, the FDA does not regulate psychotherapy, meaning that a key component of the proposed treatment fell outside of its regulatory purview>

The FDA Advisory Committee and the FDA’s most crucial critique was about functional unblinding, not about the psychotherapy+ MDMA design. FDA doesn’t regulate psychotherapy but as part of FDA’s Risk Evaluation and Mitigation Strategies (REMS), it can regulate having a licensed therapist in the room when MDMA is administered. The FDA can also require certain Furthermore, FDA doesn’t need to determine whether psychotherapy is necessary for clinical benefits, that’s too simplistic since administering MDMA outside of a therapeutic context is not what’s being asked to be approved. There are also ethical issues from giving moderate to severe PTSD patients MDMA without any therapeutic support, people could be worse off.

<Without a well-developed understanding of MDMA’s mechanisms of action, it152

is not possible to know which non-pharmacological elements of MDMA-AP are most153

essential, how these elements might be prioritised and optimised, and which154

elements (if any) might be unhelpful or even harmful (including, but not limited to,155

psychotherapeutic modality, relational stance, exposure to traumatic material,156

dosing-day support, as well as preparation provision and post-dosing meaning-157

making). These questions are, of course, based on the assumption that some158

psychotherapy is needed in MDMA treatment, a contentious point in psychedelic159

medicine research (Robinson et al, 2025).>

It’s overstated that without a well-developed understanding of MDMA’s psychological mechanisms of action, it’s not possible to study non-pharmacological elements like different psychotherapeutic approaches. There is completed and ongoing research into combining MDMA with a range of different psychotherapeutic approaches including Prolonged Exposure, Acceptance and Commitment Therapy, Cognitive Processing Therapy, Cognitive Behavioral Conjoint Therapy, Inner Directed Therapy, and Integrative MDMA-assisted Psychotherapy (see attached paper by Lerner..Yehuda, et al)

It’s clear that some of the physiological effects of MDMA can be therapeutic even in safe, recreational contexts, but can also be harmful in unsafe and unsupported contexts. One of the secondary critiques of the MDMA Phase 3 data was that 40% of the people with chronic, moderate to severe PTSD had previously used Ecstasy/MDMA. In these subjects, the mere exposure to Ecstasy/MDMA outside of a therapeutic context was not sufficiently healing of their PTSD. The methodological critique was that these people must have liked MDMA in order to volunteer for the study and were therefore more likely to have better outcomes with fewer side effects. That’s a valid critique to raise but the data showed that the 60% who had never done Ecstasy/MDMA were not statistically different in safety or efficacy than the 40% of the people who had previously tried Ecstasy/MDMA.

When patient outcomes are primary, the issue is not whether psychotherapy is needed in MDMA treatment, it’s what sort of context will be created. Will there be “psychological support”, “monitoring”, “nothing”, “therapy” and which kinds of therapy? For example, in the above cited research paper by Robinson, the subjects receiving LSD were accompanied by “dosing session monitors” who were physicians or others licensed to provide therapy, but instructed to be present to provide safety but not to provide therapy.

<To simulate aspects of the clinical use of MDMA in recent clinical188

trials (Wolfgang et al, 2025), participants will receive MDMA (initial dose, followed by189

top-up) in two sessions under clinical supervision, separated by at least one week.190

No formal psychotherapeutic procedures (e.g. those intended to direct trauma or191

experience processing) will be provided, but researchers will provide safety-oriented192

support during preparation and dosing.<

One week apart or more feels too soon. I suggest two weeks minimum between sessions for participants to learn more about the integration process, three to four weeks is even better.

What is “safety-oriented support” and how does it differ from Inner-Directed Therapy? If the therapists in this study don’t facilitate the experiential processing of emotion and memory, how will the already overly cognitive psychoanalytic psychotherapists get embodied insights into MDMA’ mechanisms of action?

You could consider adding some somatic therapists experienced in treating trauma to see what additional insights they might provide.

<• Age 25–65 years>

Why upper age limit? FDA has no upper age limit.

< Sessions 2 and 3 (dosing and acute/sub-acute data collection): This session288

includes oral administration of 80–120 mg MDMA,>

<As is typical of clinical trials, an initial307

80 mg dose will be followed by a top-up dose of 40 mg after approximately 90308

minutes if the participant consents and tolerates the lower dose well. This dosing309

protocol extends the duration of action while reducing the intensity of peak effects310

(as may be expected from a bolus dose of 120 mg). This flexibility in dosing was311

deemed appropriate by the FDA in the two largest Phase III clinical trials of MDMA312

for PTSD, in recognition of uncertainty regarding the optimal dose (Wolfgang et al,313

2025).>

Dose feels too low. There are two MDMA sessions. The first could be 80 mg followed two hours later by 40 mg. The second session could be 120 mg following by 40 or 80- I suggest 80.

<Harmful or unpleasant responses will be monitored and documented throughout the323

dosing session.>

What about positive adverse events, they should also be gathered.

It would be useful to consider adding some measures of psychological mechanisms of action added to the Phase 3 study design by Bessel van der Kolk, as reported in the attached paper. Below are the measures he used. Also attached is a 1992 paper by Dr. Charles Grob about the reflections of 20 psychiatrists on their MDMA experiences, with the abstract below.

<*The Inventory of Altered Self Capacities (IASC), is a standardized self-reported measure of an*

*individual’s psychological functioning and has been frequently utilized in treatment outcome*

*studies of PTSD [15, 40, 41]. It consists of 63 items to measure difficulties with relationships,*

*identity, and emotion regulation, rated on a 5-point Likert scale ranging from 1 (“Never”) to 5*

*(“Very Often”). The IASC consists of 11 factors and sub-factors: Items for sub-factors are*

*summed to calculate factor raw scores that range from 9 to 45 [42].*

*The Toronto Alexithymia Scale (TAS-20), is a well-validated 20-item measure of selfreported*

*difficulties with recognizing and verbalizing emotions [43]. Responses are reported*

*on a 5-point Likert scale ranging from 1 (“Strongly disagree”) to 5 (“Strongly agree”). The*

*scale is comprised of three subscales: Difficulty Describing Feelings, Difficulty Identifying*

*Feelings, and Externally-Oriented Thinking. Total scores diagnostically indicate no alexithymia*

*(�50), border-line alexithymia (51–60), and alexithymia (�61) [22].*

*The Self-Compassion Scale (SCS) is a valid and theoretically coherent self-reported measure*

*of self-compassion [44]. The SCS consists of 26 items to measure how respondents perceive*

*their own failures, suffering, or inadequacies with kindness and compassion as a part of the*

*common human experience. Respondents indicate how they often feel for each item on a*

*5-point Likert scale ranging from 1 (“Almost never”) to 5 (“Almost always”). The SCS consists*

*of six subscales: Self-Kindness, Self-Judgement, Common Humanity, Isolation, Mindfulness,*

*and Over-Identified, in which the sum of each subscale scores serves as the total score. A total*

*score of 1–2.4 indicates “low,” 2.5–3.4 “moderate,” and 3.5–5.0 “high” SCS [45].>*

*:* J Nerv Ment Dis. 1992 Jun;180(6):345-52; discussion 353-4.

doi: 10.1097/00005053-199206000-00001.

**Phenomenology and sequelae of 3,4-methylenedioxymethamphetamine use**

[M B Liester](https://pubmed.ncbi.nlm.nih.gov/?sort=date&term=Liester+MB&cauthor_id=1350613)  [^1^](https://pubmed.ncbi.nlm.nih.gov/1350613/#full-view-affiliation-1) , [C S Grob](https://pubmed.ncbi.nlm.nih.gov/?sort=date&term=Grob+CS&cauthor_id=1350613), [G L Bravo](https://pubmed.ncbi.nlm.nih.gov/?sort=date&term=Bravo+GL&cauthor_id=1350613), [R N Walsh](https://pubmed.ncbi.nlm.nih.gov/?sort=date&term=Walsh+RN&cauthor_id=1350613)

**Abstract**

3,4-Methylenedioxymethamphetamine (MDMA) has been at the center of a debate over its potential benefits as an adjunct to psychotherapy versus its capability for neurotoxic effects and is currently classified as a Schedule 1 drug by the Drug Enforcement Administration (DEA). However, as yet, there is very little methodological data on the subjective experience of the MDMA-induced state or its psychological and behavioral sequelae. The present study was, therefore, designed to obtain this kind of information. Twenty psychiatrists who had taken MDMA previously were evaluated using a semistructured interview. Subjective experience of the actual MDMA-induced state, as well as both short-term (less than 1 week) and relatively longer term (greater than 1 week) sequelae, were examined retrospectively. Side effects, insight gained, pleasure, and intensity of the MDMA experience were evaluated as were the influence of set and setting at the time the MDMA was taken and the dosage utilized. Finally, the authors discuss methodological problems and limitations of a study of this type.

< Importantly,540 participants will not be asked to discuss sensitive topics or events or trauma content541 while under the influence of MDMA, and the interviewers will redirect to present-542moment phenomenology if sensitive narratives emerge at that point.<

This may limit participants’ understanding of how MDMA impacts the experience and processing of traumatic memories.

<A qualitative, mechanism-focused617 account of MDMA’s action may clarify which elements of adjunctive psychotherapy618 are necessary, for whom, and when, thereby supporting optimisation of619 psychotherapy principles and protocols.><

Not clear this will be accomplished if subjects are redirected from engaging with traumatic memories.

This is a fascinating study that should take place.
