# Supplementary material for: Protocol for a qualitative mechanistic study of MDMA with a sample of psychoanalytic psychotherapists: A phenomenological investigation
Source: PLoS One. 2026 Jun 18;21(6):e0350728. doi: 10.1371/journal.pone.0350728 (PMC13278426; doi:10.1371/journal.pone.0350728)
Supplement: Attachment — Submitted filename: Response to Reviewers.docx [file pone.0350728.s007.docx]

Luca Aquili
Academic Editor
PLOS One

2 May 2026

Dear Dr Aquili:

Re: **PLOS One submission - *Protocol for a qualitative mechanistic study of MDMA with a sample of psychoanalytic psychotherapists: a phenomenological investigation***

Thank you very much for handling the review of our above named paper.

We have now addressed each of the reviewers’ comments and amended the paper in line with these. We have also amended some sections in which there have been trivial additional changes to the protocol since submission. These are outlined in the track changes version. We believe we have also addressed the editorial requirements for PLOS One.

**Reviewer 1**

We greatly appreciate the reviewer’s thoughtful comments and positive appraisal of our paper. We address their comments about limitations/challenges below.

Response to specific comments:

1. One challenge in the field is to bridge the psychological processes involved in psychiatric treatments with the related neurobiological processes. Although both behavioral and pharmacological treatments have a place in treatment, it is difficult to harmonize their measures, mechanisms, and interactions. In a study like this one, using a drug with known receptor actions, it might be useful to identify psychological processes that have a plausible connection to known neurobiological underpinnings, for example, those related to social attachment and bonding. This might include measuring heart rate variability or oxytocin levels.

**RESPONSE**: We thank the reviewer for highlighting this point. They are correct to notice that we do not use any objective behavioural or neurobiological indices relating to social behaviour. Although these might have been interesting additional measures, we deliberately concentrated our limited resources on prioritising high-quality qualitative data. Neurobiological assays are typically expensive and intrusive and are most informative in the context of well-powered experimental studies. Their use is also most justified when there are clear and specific hypotheses to test. In contrast our project is primarily concerned with *hypothesis generation*. Nonetheless, we recognise the value of such measures and look forward to applying them – especially in the context of dynamic therapeutic relationships – once our aspired-to psychodynamic model is developed and provides a basis for *hypothesis testing*.

2. Does MDMA have similar effects in patients and healthy volunteers? If the patients’ psychiatric conditions alter the acute neural or psychological effects of the drug, then insights from even this highly sophisticated population might be of limited value.
   With this open-label design, the participants' prior expectancies are likely to affect their responses. Will the authors be able to compare directly the participants’ expected and actual responses to the drug? Might expectances differ in those who have or have not previously used the drug?

**RESPONSE:** The reviewer highlights an important point that requires clarification. Because our study is not examining the effects of MDMA on psychopathology per se, this limitation is less of a concern for us. To reiterate, our primary goal is to use insights from psychotherapists to improve our understanding of MDMA’s potential mechanisms of action as a treatment. This, we believe, does not rely on overlapping neural or psychological processes between psychotherapists and those with psychiatric conditions. Nonetheless, because of the expert status of our participants, we fully expect them to be able to translate their insights to normal and abnormal psychological processes.

Regarding comparisons between those with/without previous experience of MDMA/psychedelics, this is not an explicit aim of the project. However, participant characteristics, including past exposure, will be reported transparently and considered as part of the whole interpretative ‘story’. We believe that considerations of context (including past exposure) will provide an opportunity to ‘thicken’ our description of participants’ experiences rather than provide a basis for direct comparisons between groups.

Regarding expectancy effects, we now clarify in the discussion that these are examined as part of our phenomena of interest, and we will examine how participants’ prior beliefs intereact with their actual MDMA experience to influence meaning-making.

Both of reviewer 1’s points made us consider whether we had been clear enough at the early stages of the paper to highlight the unique methodological approach we are taking. We therefore further foreground our approach by adding a sentence at the start of the ‘Discussion’ section of the Abstract to highlight our distinctive first-person emphasis in a specially selected participant group.

**Reviewer 2**

As above we greatly appreciate the encouraging and thoughtful comments of the reviewer.

1. The FDA Advisory Committee and the FDA’s most crucial critique was about functional unblinding, not about the psychotherapy+ MDMA design. FDA doesn’t regulate psychotherapy but as part of FDA’s Risk Evaluation and Mitigation Strategies (REMS), it can regulate having a licensed therapist in the room when MDMA is administered. The FDA can also require certain Furthermore, FDA doesn’t need to determine whether psychotherapy is necessary for clinical benefits, that’s too simplistic since administering MDMA outside of a therapeutic context is not what’s being asked to be approved. There are also ethical issues from giving moderate to severe PTSD patients MDMA without any therapeutic support, people could be worse off.

**RESPONSE**: We thank the reviewer for highlighting this. We have now corrected the relevant section of the introduction.

2. It’s overstated that without a well-developed understanding of MDMA’s psychological mechanisms of action, it’s not possible to study non-pharmacological elements like different psychotherapeutic approaches. ….

**RESPONSE**: We agree with the reviewer. We believe that his concern relates primarily to our strong statement that “….it is not possible…..”.

We have softened this definitive statement by replacing the “it is not possible” phrase with “it is difficult to establish….”

a. One week apart or more feels too soon. I suggest two weeks minimum between sessions for participants to learn more about the integration process, three to four weeks is even better.

**RESPONSE**: We agree with the reviewer that longer intervals between sessions would have been ideal. The minimum of one week was informed by stakeholder consultation which emphasised the importance of flexibility in scheduling sessions. However, while this relatively short interval applies to the two dosing sessions, there are still substantial opportunities for longer-term integration processes to occur between the second dosing session and the two follow-up sessions.

b. What is “safety-oriented support” and how does it differ from Inner-Directed Therapy? If the therapists in this study don’t facilitate the experiential processing of emotion and memory, how will the already overly cognitive psychoanalytic psychotherapists get embodied insights into MDMA’ mechanisms of action?

**RESPONSE**: In relation to the ‘safety-oriented support’ phrase, we agree its meaning is opaque. We have therefore removed this phrase completely, and instead provide a more detailed and specific outline of the nature of the interaction between researcher and participant, and clarify its limited resemblance to ‘inner directed therapy’.

c. You could consider adding some somatic therapists experienced in treating trauma to see what additional insights they might provide.

**RESPONSE**: Regarding other modalities (e.g., somatically focused therapies), although this would generate interesting and varied responses, it would be counterproductive from a ‘saturation’ perspective, which demands that such heterogeneity be minimised. This is not to say that sensory aspects of the experience will be ignored in the interviews. Indeed, it would be especially interesting if sensory and somatic aspects of the experience predominate despite the typical ‘mentalising bias’ of psychoanalytically trained therapists. Such experiences viewed through a psychoanalytic lens, we believe, would provide opportunities for novel discoveries that are less likely to occur in therapists already primed to conceptualise MDMA’s effects from a somatic perspective.

4. Why upper age limit? FDA has no upper age limit.

**RESPONSE**: We do not believe there is sufficient evidence to strongly justify any upper (or indeed, lower) age limit for MDMA. As such, any age limit can be argued to be arbitrary. We chose 65 as the ‘traditional’ cutoff separating middle and older age. Since cardiovascular disease increases with age, the risks of cardio-related adverse effects would likely also increase with age given MDMA well-established effects on heart rate and blood pressure. Given that ours is a non-clinical study, an overriding priority is participant safety, especially given that risks are not counterbalanced by any therapeutic benefits to participants.

5. Dose feels too low. There are two MDMA sessions. The first could be 80 mg followed two hours later by 40 mg. The second session could be 120 mg following by 40 or 80- I suggest 80.

**RESPONSE**: Although we agree that our proposed dosing is on the lower end of the dosing spectrum, the final dose was decided upon after thorough review of the literature, extended internal discussion within the expert team - which consists not only of leading MDMA psychopharmacologists, but also an ethicist – as well as consultation with external MDMA experts and stakeholders (psychoanalytic psychotherapists). Based on these, a maximum dose of 120 mg was deemed to appropriately balance the goal of generating the pharmacological signal of interest, while minimising the potential for significant residual symptoms. The latter was especially important in a clinician group who have ongoing patient responsibilities. We have how included an explanation of this under section 2.7.

6. What about positive adverse events, they should also be gathered.

**RESPONSE**: Although we do not conceptualise these as “positive adverse events”, we assess compassion, empathy, (epistemic) trust and positive affect (outlined in Section 2.5.2). We now also include ‘emotional breakthrough’ in our inventory of ‘positive effects’. As such, we believe there is sufficient coverage in our assessments of adverse, as well as positive, effects of MDMA.

7. It would be useful to consider adding some measures of psychological mechanisms of action added to the Phase 3 study design by Bessel van der Kolk, as reported in the attached paper. Below are the measures he used. Also attached is a 1992 paper by Dr. Charles Grob about the reflections of 20 psychiatrists on their MDMA experiences, with the abstract below.

**RESPONSE**: We thank the reviewer for this helpful suggestion. Our series of quantitative assessments already includes a number of relevant psychological constructs, including (like van der Kolk) self-compassion. However, our assessment is more inclusive than van der Kolk’s as it also incorporates ‘other-compassion’. Given our study goals (which do not relate to treating psychopathology), our measures are all tightly organised around themes relating to mentalising, trust, and attachment bonding. Given this, and given the nature of our participant group, we included several additional ‘mechanistic’ measures after piloting, relating to self-relating (other than self-compassion), ‘emotional breakthrough’ (potentially conceptualised psychoanalytically as ‘abreaction’, ‘catharsis’ or emotional insight), response to the psychedelic experience (altered consciousness and challenging experiences), as well as participants’ response to music.

8. This may limit participants’ understanding of how MDMA impacts the experience and processing of traumatic memories.

**RESPONSE**: We agree that this aspect of our protocol may limit participants’ understanding of how MDMA specifically affects trauma memories. We have now included a section in the discussion that highlights this limitation.

9. Not clear this will be accomplished if subjects are redirected from engaging with traumatic memories.

**RESPONSE**: As the reviewer will know, MDMA’s applications extend beyond PTSD treatment (e.g. social anxiety, alcohol use, and autism spectrum disorder). As such, we do not see the lack of trauma focus in our study to be especially problematic when considered alongside the potential of MDMA to treat a variety of non-trauma-related disorders. As such, we believe it is indeed realistic that our research will help us clarify “which elements of adjunctive psychotherapy are necessary, for whom, and when, thereby supporting optimisation of psychotherapy principles and protocols”.

10. This is a fascinating study that should take place.

**RESPONSE**: We greatly appreciate Dr Doblin’s kind encouragement and look forward to presenting our findings to the broader MDMA/psychedelics research community in due course.

End of ‘Response to reviewers’

**Additional changes**

Since submission we have decided that we might only include microphenomenological interviews in a proportion of participants. This decision was based on initial piloting which suggested that the routine inclusion might be overly burdensome.

We have amended the figure to be consistent with the other changes outlined here.

In some places – where we noticed omissions (e.g. from the Figure and Table, e.g. in regard to the baseline and follow-up interviews), we have included the omitted details.

Having responded thoroughly to the reviewers’ comments and amended our paper, we hope it is now acceptable for publication in PLOS One.

Many thanks again for handling our submission. We look forward to hearing the outcome soon.

Sincerely,

Sunjeev Kamboj, PhD

**Professor of Translational Clinical Psychology**

**University College London**
