# Supplementary material for: Protocol for a qualitative mechanistic study of MDMA with a sample of psychoanalytic psychotherapists: A phenomenological investigation
Source: PLoS One. 2026 Jun 18;21(6):e0350728. doi: 10.1371/journal.pone.0350728 (PMC13278426; doi:10.1371/journal.pone.0350728)
Supplement: Peer review history

# Peer review: Decision Letter 0

**DOI:** 10.1371/journal.pone.0350728.r001

**Authors:** Luca Aquili

13 Apr 2026

PONE-D-26-08741

Protocol for a qualitative mechanistic study of MDMA with a sample of psychoanalytic psychotherapists: a phenomenological investigation

PLOS One

Dear Dr. Kamboj

Thank you for submitting your manuscript to PLOS ONE. After careful consideration, we feel that it has merit but does not fully meet PLOS ONE’s publication criteria as it currently stands. Therefore, we invite you to submit a revised version of the manuscript that addresses the points raised during the review process.

We would be grateful if you could address the comments raised by the two reviewers in Sections, 2, 3 and 6 of this email and the related attachments.

Please submit your revised manuscript by May 28 2026 11:59PM. If you will need more time than this to complete your revisions, please reply to this message or contact the journal office at plosone@plos.org. When you're ready to submit your revision, log on to https://www.editorialmanager.com/pone/ and select the 'Submissions Needing Revision' folder to locate your manuscript file.

Please include the following items when submitting your revised manuscript:

- A letter that responds to each point raised by the academic editor and reviewer(s). You should upload this letter as a separate file labeled 'Response to Reviewers'.
- A marked-up copy of your manuscript that highlights changes made to the original version. You should upload this as a separate file labeled 'Revised Manuscript with Track Changes'.
- An unmarked version of your revised paper without tracked changes. You should upload this as a separate file labeled 'Manuscript'.

If you would like to make changes to your financial disclosure, please include your updated statement in your cover letter. Guidelines for resubmitting your figure files are available below the reviewer comments at the end of this letter.

If applicable, we recommend that you deposit your laboratory protocols in protocols.io to enhance the reproducibility of your results. Protocols.io assigns your protocol its own identifier (DOI) so that it can be cited independently in the future. For instructions see: https://journals.plos.org/plosone/s/submission-guidelines#loc-laboratory-protocols. Additionally, PLOS ONE offers an option for publishing peer-reviewed Lab Protocol articles, which describe protocols hosted on protocols.io. Read more information on sharing protocols at https://plos.org/protocols?utm_medium=editorial-email&utm_source=authorletters&utm_campaign=protocols.

As the corresponding author, your ORCID iD is verified in the submission system and will appear in the published article. PLOS supports the use of ORCID, and we encourage all coauthors to register for an ORCID iD and use it as well. Please encourage your coauthors to verify their ORCID iD within the submission system before final acceptance, as unverified ORCID iDs will not appear in the published article. *Only*   the individual author can complete the verification step; PLOS staff *cannot*   verify ORCID iDs on behalf of authors.

We look forward to receiving your revised manuscript.

Kind regards,

Luca Aquili

Academic Editor

PLOS One

Journal requirements:

When submitting your revision, we need you to address these additional requirements.

1. Please ensure that your manuscript meets PLOS ONE's style requirements, including those for file naming. The PLOS ONE style templates can be found at

https://journals.plos.org/plosone/s/file?id=wjVg/PLOSOne_formatting_sample_main_body.pdf and

https://journals.plos.org/plosone/s/file?id=ba62/PLOSOne_formatting_sample_title_authors_affiliations.pdf

2. Your ethics statement should only appear in the Methods section of your manuscript. If your ethics statement is written in any section besides the Methods, please move it to the Methods section and delete it from any other section. Please ensure that your ethics statement is included in your manuscript, as the ethics statement entered into the online submission form will not be published alongside your manuscript. If the reviewer comments include a recommendation to cite specific previously published works, please review and evaluate these publications to determine whether they are relevant and should be cited. There is no requirement to cite these works unless the editor has indicated otherwise.

Please review your reference list to ensure that it is complete and correct. If you have cited papers that have been retracted, please include the rationale for doing so in the manuscript text, or remove these references and replace them with relevant current references. Any changes to the reference list should be mentioned in the rebuttal letter that accompanies your revised manuscript. If you need to cite a retracted article, indicate the article’s retracted status in the References list and also include a citation and full reference for the retraction notice.

[Note: HTML markup is below. Please do not edit.]

Reviewers' comments:

Reviewer's Responses to Questions

**Comments to the Author**

1. Does the manuscript provide a valid rationale for the proposed study, with clearly identified and justified research questions?

The research question outlined is expected to address a valid academic problem or topic and contribute to the base of knowledge in the field.

Reviewer #1: Yes

Reviewer #2: Yes

**********

2. Is the protocol technically sound and planned in a manner that will lead to a meaningful outcome and allow testing the stated hypotheses?

The manuscript should describe the methods in sufficient detail to prevent undisclosed flexibility in the experimental procedure or analysis pipeline, including sufficient outcome-neutral conditions (e.g. necessary controls, absence of floor or ceiling effects) to test the proposed hypotheses and a statistical power analysis where applicable. As there may be aspects of the methodology and analysis which can only be refined once the work is undertaken, authors should outline potential assumptions and explicitly describe what aspects of the proposed analyses, if any, are exploratory.

Reviewer #1: Yes

Reviewer #2: Yes

**********

3. Is the methodology feasible and described in sufficient detail to allow the work to be replicable?

Descriptions of methods and materials in the protocol should be reported in sufficient detail for another researcher to reproduce all experiments and analyses. The protocol should describe the appropriate controls, sample size calculations, and replication needed to ensure that the data are robust and reproducible.

Reviewer #1: Yes

Reviewer #2: Yes

**********

4. Have the authors described where all data underlying the findings will be made available when the study is complete?

The PLOS Data policy requires authors to make all data underlying the findings described in their manuscript fully available without restriction, with rare exception, at the time of publication. The data should be provided as part of the manuscript or its supporting information, or deposited to a public repository. For example, in addition to summary statistics, the data points behind means, medians and variance measures should be available. If there are restrictions on publicly sharing data—e.g. participant privacy or use of data from a third party—those must be specified.

Reviewer #1: Yes

Reviewer #2: Yes

**********

5. Is the manuscript presented in an intelligible fashion and written in standard English?

PLOS ONE does not copyedit accepted manuscripts, so the language in submitted articles must be clear, correct, and unambiguous. Any typographical or grammatical errors should be corrected at revision, so please note any specific errors here.

Reviewer #1: Yes

Reviewer #2: Yes

**********

6. Review Comments to the Author

Please use the space provided to explain your answers to the questions above and, if applicable, provide comments about issues authors must address before this protocol can be accepted for publication. You may also include additional comments for the author, including concerns about research or publication ethics.

You may also provide optional suggestions and comments to authors that they might find helpful in planning their study.

(Please upload your review as an attachment if it exceeds 20,000 characters)

Reviewer #1: This paper describes a proposed study to characterize the psychological effects of MDMA in a sample of experienced psychoanalysts. The rationale for using this population is that they are expected to be uniquely attuned to psychological (emotional and relational) processes that may form the basis of the therapeutic value of MDMA in psychiatric treatment.

The manuscript, and the protocol, have many strengths. The proposed study is clearly described, and it is carefully designed with appropriate outcome measures. The main claim of the paper is that this psychologically sophisticated sample of participants will provide new insights about how MDMA changes behavior in patients. Such new insights would be of great value to the discipline, as it struggles to identify processes and measures that mediate therapeutic benefits of psychedelic drugs. The doses, dosing interval, sample size, and broad range of outcome measures have been thoughtfully developed. The proposed outcome measures are appropriate in light of what is known from controlled MDMA studies so far, together with accumulated research on the processes involved in psychoanalytic or related psychiatric treatments. With a qualitative and descriptive study of this kind, there is no clear hypothesis, but the findings may provide the basis for developing operational measures in future studies.

Here are some minor general comments and questions.

One challenge in the field is to bridge the psychological processes involved in psychiatric treatments with the related neurobiological processes. Although both behavioral and pharmacological treatments have a place in treatment, it is difficult to harmonize their measures, mechanisms, and interactions. In a study like this one, using a drug with known receptor actions, it might be useful to identify psychological processes that have a plausible connection to known neurobiological underpinnings, for example those related to social attachment and bonding. This might include measuring heart rate variability or oxytocin levels.

Does MDMA have similar effects in patients and healthy volunteers? If the patients’ psychiatric conditions alter the acute neural or psychological effects of the drug, then insights from even this highly sophisticated population might be of limited value.

With this open label design, the participants prior expectancies are likely to affect their responses. Will the authors be able to compare directly the participants’ expected and actual responses to the drug? Might expectances differ in those who have or have not previously used the drug?

Reviewer #2: I've uploaded my comments in a Word document with sections from the paper and then my comments afterwards.

**********

7. PLOS authors have the option to publish the peer review history of their article (what does this mean?). If published, this will include your full peer review and any attached files.

If you choose “no”, your identity will remain anonymous but your review may still be made public.

**Do you want your identity to be public for this peer review?**   For information about this choice, including consent withdrawal, please see our Privacy Policy.

Reviewer #1: No

Reviewer #2: **Yes:**   Richard Doblin, Ph.D.

**********

[NOTE: If reviewer comments were submitted as an attachment file, they will be attached to this email and accessible via the submission site. Please log into your account, locate the manuscript record, and check for the action link "View Attachments". If this link does not appear, there are no attachment files.]

To ensure your figures meet our technical requirements, please review our figure guidelines: https://journals.plos.org/plosone/s/figures

You may also use PLOS’s free figure tool, NAAS, to help you prepare publication quality figures: https://journals.plos.org/plosone/s/figures#loc-tools-for-figure-preparation.

NAAS will assess whether your figures meet our technical requirements by comparing each figure against our figure specifications.

Attachment Submitted filename: Bessels paper published.pdf

Attachment Submitted filename: Grob PsychiatristsMDMA.pdf

Attachment Submitted filename: lehrnerYehuda2026structured-protocols-in-mdma.pdf

Attachment Submitted filename: My Comments on MDMA in psychoanalytic psychotherapists.docx

# Author comment: Author response to Decision Letter 1

**DOI:** 10.1371/journal.pone.0350728.r002

11 May 2026

Reviewer 1

We greatly appreciate the reviewer’s thoughtful comments and positive appraisal of our paper. We address their comments about limitations/challenges below.

Response to specific comments:

1. One challenge in the field is to bridge the psychological processes involved in psychiatric treatments with the related neurobiological processes. Although both behavioral and pharmacological treatments have a place in treatment, it is difficult to harmonize their measures, mechanisms, and interactions. In a study like this one, using a drug with known receptor actions, it might be useful to identify psychological processes that have a plausible connection to known neurobiological underpinnings, for example, those related to social attachment and bonding. This might include measuring heart rate variability or oxytocin levels.

RESPONSE: We thank the reviewer for highlighting this point. They are correct to notice that we do not use any objective behavioural or neurobiological indices relating to social behaviour. Although these might have been interesting additional measures, we deliberately concentrated our limited resources on prioritising high-quality qualitative data. Neurobiological assays are typically expensive and intrusive and are most informative in the context of well-powered experimental studies. Their use is also most justified when there are clear and specific hypotheses to test. In contrast our project is primarily concerned with *hypothesis generation*. Nonetheless, we recognise the value of such measures and look forward to applying them – especially in the context of dynamic therapeutic relationships – once our aspired-to psychodynamic model is developed and provides a basis for *hypothesis testing*.

2. Does MDMA have similar effects in patients and healthy volunteers? If the patients’ psychiatric conditions alter the acute neural or psychological effects of the drug, then insights from even this highly sophisticated population might be of limited value.

With this open-label design, the participants' prior expectancies are likely to affect their responses. Will the authors be able to compare directly the participants’ expected and actual responses to the drug? Might expectances differ in those who have or have not previously used the drug?

RESPONSE: The reviewer highlights an important point that requires clarification. Because our study is not examining the effects of MDMA on psychopathology per se, this limitation is less of a concern for us. To reiterate, our primary goal is to use insights from psychotherapists to improve our understanding of MDMA’s potential mechanisms of action as a treatment. This, we believe, does not rely on overlapping neural or psychological processes between psychotherapists and those with psychiatric conditions. Nonetheless, because of the expert status of our participants, we fully expect them to be able to translate their insights to normal and abnormal psychological processes.

Regarding comparisons between those with/without previous experience of MDMA/psychedelics, this is not an explicit aim of the project. However, participant characteristics, including past exposure, will be reported transparently and considered as part of the whole interpretative ‘story’. We believe that considerations of context (including past exposure) will provide an opportunity to ‘thicken’ our description of participants’ experiences rather than provide a basis for direct comparisons between groups.

Regarding expectancy effects, we now clarify in the discussion that these are examined as part of our phenomena of interest, and we will examine how participants’ prior beliefs intereact with their actual MDMA experience to influence meaning-making.

Both of reviewer 1’s points made us consider whether we had been clear enough at the early stages of the paper to highlight the unique methodological approach we are taking. We therefore further foreground our approach by adding a sentence at the start of the ‘Discussion’ section of the Abstract to highlight our distinctive first-person emphasis in a specially selected participant group.

Reviewer 2

As above we greatly appreciate the encouraging and thoughtful comments of the reviewer.

1. The FDA Advisory Committee and the FDA’s most crucial critique was about functional unblinding, not about the psychotherapy+ MDMA design. FDA doesn’t regulate psychotherapy but as part of FDA’s Risk Evaluation and Mitigation Strategies (REMS), it can regulate having a licensed therapist in the room when MDMA is administered. The FDA can also require certain Furthermore, FDA doesn’t need to determine whether psychotherapy is necessary for clinical benefits, that’s too simplistic since administering MDMA outside of a therapeutic context is not what’s being asked to be approved. There are also ethical issues from giving moderate to severe PTSD patients MDMA without any therapeutic support, people could be worse off.

RESPONSE: We thank the reviewer for highlighting this. We have now corrected the relevant section of the introduction.

2. It’s overstated that without a well-developed understanding of MDMA’s psychological mechanisms of action, it’s not possible to study non-pharmacological elements like different psychotherapeutic approaches. ….

RESPONSE: We agree with the reviewer. We believe that his concern relates primarily to our strong statement that “….it is not possible…..”.

We have softened this definitive statement by replacing the “it is not possible” phrase with “it is difficult to establish….”

3.

a. One week apart or more feels too soon. I suggest two weeks minimum between sessions for participants to learn more about the integration process, three to four weeks is even better.

RESPONSE: We agree with the reviewer that longer intervals between sessions would have been ideal. The minimum of one week was informed by stakeholder consultation which emphasised the importance of flexibility in scheduling sessions. However, while this relatively short interval applies to the two dosing sessions, there are still substantial opportunities for longer-term integration processes to occur between the second dosing session and the two follow-up sessions.

b. What is “safety-oriented support” and how does it differ from Inner-Directed Therapy? If the therapists in this study don’t facilitate the experiential processing of emotion and memory, how will the already overly cognitive psychoanalytic psychotherapists get embodied insights into MDMA’ mechanisms of action?

RESPONSE: In relation to the ‘safety-oriented support’ phrase, we agree its meaning is opaque. We have therefore removed this phrase completely, and instead provide a more detailed and specific outline of the nature of the interaction between researcher and participant, and clarify its limited resemblance to ‘inner directed therapy’.

c. You could consider adding some somatic therapists experienced in treating trauma to see what additional insights they might provide.

RESPONSE: Regarding other modalities (e.g., somatically focused therapies), although this would generate interesting and varied responses, it would be counterproductive from a ‘saturation’ perspective, which demands that such heterogeneity be minimised. This is not to say that sensory aspects of the experience will be ignored in the interviews. Indeed, it would be especially interesting if sensory and somatic aspects of the experience predominate despite the typical ‘mentalising bias’ of psychoanalytically trained therapists. Such experiences viewed through a psychoanalytic lens, we believe, would provide opportunities for novel discoveries that are less likely to occur in therapists already primed to conceptualise MDMA’s effects from a somatic perspective.

4. Why upper age limit? FDA has no upper age limit.

RESPONSE: We do not believe there is sufficient evidence to strongly justify any upper (or indeed, lower) age limit for MDMA. As such, any age limit can be argued to be arbitrary. We chose 65 as the ‘traditional’ cutoff separating middle and older age. Since cardiovascular disease increases with age, the risks of cardio-related adverse effects would likely also increase with age given MDMA well-established effects on heart rate and blood pressure. Given that ours is a non-clinical study, an overriding priority is participant safety, especially given that risks are not counterbalanced by any therapeutic benefits to participants.

5. Dose feels too low. There are two MDMA sessions. The first could be 80 mg followed two hours later by 40 mg. The second session could be 120 mg following by 40 or 80- I suggest 80.

RESPONSE: Although we agree that our proposed dosing is on the lower end of the dosing spectrum, the final dose was decided upon after thorough review of the literature, extended internal discussion within the expert team - which consists not only of leading MDMA psychopharmacologists, but also an ethicist – as well as consultation with external MDMA experts and stakeholders (psychoanalytic psychotherapists). Based on these, a maximum dose of 120 mg was deemed to appropriately balance the goal of generating the pharmacological signal of interest, while minimising the potential for significant residual symptoms. The latter was especially important in a clinician group who have ongoing patient responsibilities. We have how included an explanation of this under section 2.7.

6. What about positive adverse events, they should also be gathered.

RESPONSE: Although we do not conceptualise these as “positive adverse events”, we assess compassion, empathy, (epistemic) trust and positive affect (outlined in Section 2.5.2). We now also include ‘emotional breakthrough’ in our inventory of ‘positive effects’. As such, we believe there is sufficient coverage in our assessments of adverse, as well as positive, effects of MDMA.

7. It would be useful to consider adding some measures of psychological mechanisms of action added to the Phase 3 study design by Bessel van der Kolk, as reported in the attached paper. Below are the measures he used. Also attached is a 1992 paper by Dr. Charles Grob about the reflections of 20 psychiatrists on their MDMA experiences, with the abstract below.

RESPONSE: We thank the reviewer for this helpful suggestion. Our series of quantitative assessments already includes a number of relevant psychological constructs, including (like van der Kolk) self-compassion. However, our assessment is more inclusive than van der Kolk’s as it also incorporates ‘other-compassion’. Given our study goals (which do not relate to treating psychopathology), our measures are all tightly organised around themes relating to mentalising, trust, and attachment bonding. Given this, and given the nature of our participant group, we included several additional ‘mechanistic’ measures after piloting, relating to self-relating (other than self-compassion), ‘emotional breakthrough’ (potentially conceptualised psychoanalytically as ‘abreaction’, ‘catharsis’ or emotional insight), response to the psychedelic experience (altered consciousness and challenging experiences), as well as participants’ response to music.

8. This may limit participants’ understanding of how MDMA impacts the experience and processing of traumatic memories.

RESPONSE: We agree that this aspect of our protocol may limit participants’ understanding of how MDMA specifically affects trauma memories. We have now included a section in the discussion that highlights this limitation.

9. Not clear this will be accomplished if subjects are redirected from engaging with traumatic memories.

RESPONSE: As the reviewer will know, MDMA’s applications extend beyond PTSD treatment (e.g. social anxiety, alcohol use, and autism spectrum disorder). As such, we do not see the lack of trauma focus in our study to be especially problematic when considered alongside the potential of MDMA to treat a variety of non-trauma-related disorders. As such, we believe it is indeed realistic that our research will help us clarify “which elements of adjunctive psychotherapy are necessary, for whom, and when, thereby supporting optimisation of psychotherapy principles and protocols”.

10. This is a fascinating study that should take place.

RESPONSE: We greatly appreciate Dr Doblin’s kind encouragement and look forward to presenting our findings to the broader MDMA/psychedelics research community in due course.

Attachment Submitted filename: Response to Reviewers.docx

# Peer review: Decision Letter 1

**DOI:** 10.1371/journal.pone.0350728.r003

**Authors:** Luca Aquili

19 May 2026

Protocol for a qualitative mechanistic study of MDMA with a sample of psychoanalytic psychotherapists: a phenomenological investigation

PONE-D-26-08741R1

Dear Dr. Kamboj,

We’re pleased to inform you that your manuscript has been judged scientifically suitable for publication and will be formally accepted for publication once it meets all outstanding technical requirements.

Within one week, you’ll receive an e-mail detailing the required amendments. When these have been addressed, you’ll receive a formal acceptance letter and your manuscript will be scheduled for publication.

An invoice will be generated when your article is formally accepted. Please note, if your institution has a publishing partnership with PLOS and your article meets the relevant criteria, all or part of your publication costs will be covered. Please make sure your user information is up-to-date by logging into Editorial Manager at Editorial Manager® and clicking the ‘Update My Information' link at the top of the page. For questions related to billing, please contact billing support.

If your institution or institutions have a press office, please notify them about your upcoming paper to help maximize its impact. If they’ll be preparing press materials, please inform our press team as soon as possible -- no later than 48 hours after receiving the formal acceptance. Your manuscript will remain under strict press embargo until 2 pm Eastern Time on the date of publication. For more information, please contact onepress@plos.org.

Kind regards,

Luca Aquili

Academic Editor

PLOS One

Additional Editor Comments (optional):

Reviewers' comments:

Reviewer's Responses to Questions

**Comments to the Author**

1. Does the manuscript provide a valid rationale for the proposed study, with clearly identified and justified research questions?

The research question outlined is expected to address a valid academic problem or topic and contribute to the base of knowledge in the field.

Reviewer #2: Yes

**********

2. Is the protocol technically sound and planned in a manner that will lead to a meaningful outcome and allow testing the stated hypotheses?

The manuscript should describe the methods in sufficient detail to prevent undisclosed flexibility in the experimental procedure or analysis pipeline, including sufficient outcome-neutral conditions (e.g. necessary controls, absence of floor or ceiling effects) to test the proposed hypotheses and a statistical power analysis where applicable. As there may be aspects of the methodology and analysis which can only be refined once the work is undertaken, authors should outline potential assumptions and explicitly describe what aspects of the proposed analyses, if any, are exploratory.

Reviewer #2: Yes

**********

3. Is the methodology feasible and described in sufficient detail to allow the work to be replicable?

Descriptions of methods and materials in the protocol should be reported in sufficient detail for another researcher to reproduce all experiments and analyses. The protocol should describe the appropriate controls, sample size calculations, and replication needed to ensure that the data are robust and reproducible.

Reviewer #2: Yes

**********

4. Have the authors described where all data underlying the findings will be made available when the study is complete?

The PLOS Data policy requires authors to make all data underlying the findings described in their manuscript fully available without restriction, with rare exception, at the time of publication. The data should be provided as part of the manuscript or its supporting information, or deposited to a public repository. For example, in addition to summary statistics, the data points behind means, medians and variance measures should be available. If there are restrictions on publicly sharing data—e.g. participant privacy or use of data from a third party—those must be specified.

Reviewer #2: Yes

**********

5. Is the manuscript presented in an intelligible fashion and written in standard English?

PLOS ONE does not copyedit accepted manuscripts, so the language in submitted articles must be clear, correct, and unambiguous. Any typographical or grammatical errors should be corrected at revision, so please note any specific errors here.

Reviewer #2: Yes

**********

6. Review Comments to the Author

Please use the space provided to explain your answers to the questions above and, if applicable, provide comments about issues authors must address before this protocol can be accepted for publication. You may also include additional comments for the author, including concerns about research or publication ethics.

You may also provide optional suggestions and comments to authors that they might find helpful in planning their study.

(Please upload your review as an attachment if it exceeds 20,000 characters)

Reviewer #2: I'm fine with the paper being published as is with no further changes. However, I am unpersuaded by several responses to my comments in my previous review. There is sufficient cardiovascular screening to enroll people over age 65, with that age limit potentially excluding some of the most experienced psychodynamically trained psychotherapists. The doses of MDMA, 80 mg followed by 40 mg, are lower than used in most therapy research which often includes doses of 120 mg followed by 60 mg, and thus the study may miss gathering some fundamentally important data. The idea that it's important to avoid "a rapid peak effect (as might be expected from a 120 mg bolus)" is not based on data and is not a significant factor in generating adverse events. I suggest giving subjects the option to increase the dose in their second experience to 120 mg followed by 40 mg (or if you are really bold, followed by 80 mg. I also am unpersuaded that dosing people one week apart is a good idea "to provide greater flexibility to participants." The idea that the participants should be able to get back to work with their patient responsibilities further limits the data that can be gathered. If the subjects are not wanting to let the experience deepen since they think they need to be ready to see patients the next day or in two days, the experience can be more superficial. (I hope the participants are being instructed that they should have no responsibilities the day after the MDMA experience, and if they need more time to integrate, they should have a plan for a colleague to take over for them till they feel grounded again). I am profoundly unpersuaded that if participants start to process prior traumas, that they should be redirected to "to present moment phenomenology if sensitive narratives emerge..." The authors of the paper make a big deal about how experienced psychodynamically trained psychotherapists are able to understand and describe their internal states and understand about transference and countertransference. The fear that such experienced psychotherapists would be destabilized by addressing their own trauma is contradicted by their supposed experience with their own psychotherapy of several times a week for several years. The paper says, "the team considered it ethically problematic to encourage participants to engage with and discuss traumatic materials when they might not, ordinarily, be inclined to do so (i.e. outside of the MDMA state)".Has the team considered the ethical problems in having participants recall traumatic memories under the influence of MDMA and then being redirected away from those memories? The paper says, "Equally, avoiding trauma processing will enable us to preserve the interpretability of mechanisms because we will not be conflating drug effects with exposure-based therapeutic processes". What this avoidance can miss is that MDMA's drug effects help people engage with "exposure-based therapeutic processes." The US Dept. of Defense has funded a $4.9 million study to examine combining MDMA with Prolonged Exposure,specifically to look at how MDMA drug effects can potentially aid in "exposure-based therapeutic processes." Pilot studies have already demonstrated that MDMA can help subjects engage with PE, when without MDMA about 50% of subjects drop out of PE (and also CPT). The paper also says, "As the reviewer will know, MDMA’s applications extend beyond PTSD treatment (e.g. social anxiety, alcohol use, and autism spectrum disorder). As such, we do not see the lack of trauma focus in our study to be especially problematic when considered alongside the potential of MDMA to treat a variety of non-trauma-related disorders" . As the authors should know, in the study in England looking into MDMA-assisted therapy to treat AUD, most of the participants ended up dealing with unprocessed trauma that led them to run away from their emotions with alcohol. Society anxiety is often connected to various fears and sometimes past traumas so the idea that only PTSD is based on trauma is fundamentally wrong. This is a fascinating study that I worry will not generate as rich of a data set as it could, due to several risk-averse decisions and trade-offs. Still, I'm highly supportive of this study and this paper. I've attached a paper by Amy Lerner and Rachel Yehuda about the trade-offs between patient-centered therapies and structured therapies like PE and CPT and CBT.

**********

7. PLOS authors have the option to publish the peer review history of their article (what does this mean?). If published, this will include your full peer review and any attached files.

If you choose “no”, your identity will remain anonymous but your review may still be made public.

**Do you want your identity to be public for this peer review?**   For information about this choice, including consent withdrawal, please see our Privacy Policy.

Reviewer #2: **Yes:**   Richard Doblin

**********

Attachment Submitted filename: lehrnerYehuda2026structured-protocols-in-mdma.pdf

# Editor report: Acceptance letter

**DOI:** 10.1371/journal.pone.0350728.r004

**Authors:** Luca Aquili

PONE-D-26-08741R1

PLOS One

Dear Dr. Kamboj,

I'm pleased to inform you that your manuscript has been deemed suitable for publication in PLOS One. Congratulations! Your manuscript is now being handed over to our production team.

At this stage, our production department will prepare your paper for publication. This includes ensuring the following:

* All references, tables, and figures are properly cited

* All relevant supporting information is included in the manuscript submission,

* There are no issues that prevent the paper from being properly typeset

You will receive further instructions from the production team, including instructions on how to review your proof when it is ready. Please keep in mind that we are working through a large volume of accepted articles, so please give us a few days to review your paper and let you know the next and final steps.

Lastly, if your institution or institutions have a press office, please let them know about your upcoming paper now to help maximize its impact. If they'll be preparing press materials, please inform our press team within the next 48 hours. Your manuscript will remain under strict press embargo until 2 pm Eastern Time on the date of publication. For more information, please contact onepress@plos.org.

You will receive an invoice from PLOS for your publication fee after your manuscript has reached the completed accept phase. If you receive an email requesting payment before acceptance or for any other service, this may be a phishing scheme. Learn how to identify phishing emails and protect your accounts at https://explore.plos.org/phishing.

If we can help with anything else, please email us at customercare@plos.org.

Thank you for submitting your work to PLOS ONE and supporting open access.

Kind regards,

PLOS ONE Editorial Office Staff

on behalf of

Dr. Luca Aquili

Academic Editor

PLOS One
